# Measurement properties of cervical joint position error in people with and without neck pain: a systematic review and narrative synthesis

**DOI:** 10.1186/s12891-023-07111-4

**Published:** 2024-01-10

**Authors:** Ahmad AlDahas, Nicola R. Heneghan, Shouq Althobaiti, Janet A. Deane, Alison Rushton, Deborah Falla

**Affiliations:** 1 Centre of Precision Rehabilitation for Spinal Pain (CPR Spine), School of Sport, Exercise and Rehabilitation Sciences, College of Life and Environmental Sciences, University of Birmingham, Edgbaston, Birmingham, B15 2TT UK; 2 Department of Physical Therapy, Al-Sabah Medical Hospital, Ministry of Public Health, Kuwait City, Kuwait; 3 School of Physical Therapy, Western University, London, ON Canada

**Keywords:** Neck pain, Proprioception, Sensorimotor, Position sense, Position error, Measurement properties

## Abstract

**Introduction:**

Proprioception can be impaired in people with neck pain. The cervical joint position sense test, which measures joint position error (JPE), is the most common test used to assess neck proprioception. The aim of this systematic review was to assess the measurement properties of this test for the assessment of people with and without neck pain.

**Methods:**

This systematic review was registered prospectively on Prospero (CRD42020188715). It was designed using the COSMIN guidelines and reported in line with the PRISMA checklist. Two reviewers independently searched Medline, Embase, SportDiscus, and CINAHL Plus databases from inception to the 24th July 2022 with an update of the search conducted until 14th of October 2023. The COSMIN risk of bias checklist was used to assess the risk of bias in each study. The updated criteria for good measurement properties were used to rate individual studies and then the overall pooled results. The level of evidence was rated by two reviewers independently using a modified GRADE approach.

**Results:**

Fifteen studies were included in this review, 13 reporting absolute JPE and 2 reporting constant JPE. The measurement properties assessed were reliability, measurement error, and validity. The measurement of JPE showed sufficient reliability and validity, however, the level of evidence was low/very low for both measurement properties, apart from convergent validity of the constant JPE, which was high.

**Conclusion:**

The measure of cervical JPE showed sufficient reliability and validity but with low/very low levels of evidence. Further studies are required to investigate the reliability and validity of this test as well as the responsiveness of the measure.

**Supplementary Information:**

The online version contains supplementary material available at 10.1186/s12891-023-07111-4.

## Background

Cervical sensorimotor control relies on the integration of visual, vestibular, and somatosensory information [1]. Afferent information from muscle spindles is known to contribute significantly to cervical proprioception; in particular muscles in the upper cervical region contain a high density of muscle spindles, which implies that they have an important role in neck proprioception [2].

Several outcome measures have been used to assess cervical proprioception with the joint position sense test being the most common test to evaluate joint position error (JPE) [2]. The joint position sense test determines a person’s ability to relocate their head back to a target position following active neck movement whilst their vision is occluded. Two commonly used joint position sense tests that measure JPE include the neutral head position (NHP) test, having the participant return to a neutral head position following active movement, or the target head position (THP) test, where target head position is determined by the participant or assessor [3].

Several studies have evaluated cervical proprioception by quantifying JPE in people with neck pain and have shown that cervical proprioception can be impaired in people with neck pain regardless of the aetiology [4–6]. For example, Revel et al. reported a higher repositioning error in people with chronic neck pain (CNP) after returning to neutral from flexion, extension, and right and left rotation when compared to asymptomatic participants [4]. Feipel et al. showed that people with chronic whiplash-associated disorders (WAD) had higher repositioning errors compared to asymptomatic participants [7]. Nevertheless, evidence indicates that cervical proprioception is more likely to be affected and to a greater extent in people that have trauma-induced neck pain [8]. Additionally, people with WAD and dizziness complaints usually have higher repositioning errors when compared to people with WAD but without dizziness [9, 10]. Impaired neck proprioception is thought to be at least partially attributed to a disturbance in cervical afferent activity [2]. Several mechanisms can contribute to this disturbance such as direct trauma to cervical structures, the influence of nociception, the presence of inflammatory mediators [9], and psychological distress [8]. A disturbance in cervical afferent input is also thought to contribute symptoms of dizziness for some patients [8].

Knowledge of the psychometric properties of outcome measures, which includes their reliability, validity, and responsiveness, are important as they reflect data accuracy and precision [11]. Michiels et al. carried out a systematic review investigating the measurement properties of cervical sensorimotor control tests [12]. In their 2012 review, they investigated the reliability and discriminative validity of tests. Although this systematic review did not use the now recommended Consensus-based Standards for the Selection of health Measurement Instrument (COSMIN) reporting guidelines [13], they reported that the NHP test showed fair to excellent reliability (ICC range: 0.35–0.87) while the THP showed poor to excellent reliability (ICC range: 0.01–0.9). Additionally, the JPE test was able to discriminate between people with and without chronic neck pain.

Given the number of publications since this last review, in this current systematic review, we aimed to build upon this research to synthesise the available evidence in relation to a range of measurement properties (reliability, measurement error, validity, and responsiveness) of the measure of cervical JPE for the assessment of people with and without neck pain.

## Design and methods

This systematic review was designed using the COSMIN risk of bias (RoB) guidelines for reliability and measurement error of outcome measurement instruments as well as the COSMIN methodology for systematic reviews of Patient-Reported Outcome Measures (PROMs) [13, 14] and is reported in accordance with the Preferred Reporting Items for Systematic Review and Meta-Analysis (PRISMA) [15]. The protocol was registered with PROSPERO on the 10th of July 2020 (CRD42020188715).

### Deviations from the study protocol

The initial protocol described a systematic review of the measurement properties of proprioception tests for all regions of the spine. However, following an initial review of the literature and appreciation of the number of studies conducted in different spinal regions, the decision was made to focus on the measurement properties of cervical JPE only. Additionally, the original plan was to use the COSMIN RoB checklist for PROMs, however since publishing the protocol, the authors were made aware of the new COSMIN RoB checklist for reliability and measurement error of outcome measurement instruments. Thus, this new tool was used to assess RoB of reliability, measurement error, and criterion validity [14]. The COSMIN RoB checklist for PROMs was used to assess construct and discriminative validity [16] as suggested in the manual for the COSMIN RoB checklist for reliability and measurement error of outcome measurement instruments.

### Eligibility criteria

The following inclusion criteria are based on the Sample, Phenomena of Interest, Design, Evaluation, and Research type (SPIDER) guidelines [17].

- Sample: people with and without neck pain aged ≥ 18 years. Those with neck pain included regardless of the stage of their neck pain (e.g., acute, or chronic) or aetiology (e.g., non-specific or attributed to pathology).
- Phenomena of interest: cervical proprioception.
- Design: any study which investigated at least one of the domains (reliability, validity, responsiveness, and their sub-domains) of the COSMIN checklist and reported absolute error (AE) or constant error (CE) in degrees.
- Evaluation: any study that evaluated measurement properties of the measure of cervical JPE.
- Research type: quantitative research.

### Exclusion criteria

Studies that included patients that had undergone cervical spine surgery and studies not written in English were excluded.

### Information sources

The following databases were searched as recommended by the COSMIN guidelines for systematic reviews [13], from inception to the 24th July 2022 with an update of the search conducted until 14th of October 2023: MEDLINE, Embase, SportDiscus, and CINAHL plus. Manual searches were carried out for: The Spine Journal, European Spine Journal, Journal of Musculoskeletal Science and Practice, and the Journal of Orthopaedic and Sport Physical Therapy. Grey literature (Open Grey, ProQuest, and EThOS) was hand searched.

### Search strategy

Following scoping searches and discussions with co-authors, the search strategy was developed, and a librarian was consulted. Search terms are provided in Table 1. Search syntax was translated to meet the requirements of each database.

**Table 1:** MEDLINE syntax used in MEDLINE database

**Search terms**	Neck pain OR neck dysfunction OR cervical pain OR cervical dysfunction AND Propriocept* OR movement sense OR kinesthes* OR repositioning OR repositioning error OR position sense OR motion perception OR active position sense OR passive position sense AND Reliability OR validity OR responsiveness OR reproducibility of results OR reproducib* OR reliab* OR valid* OR stability OR interrater OR interrater OR intrarater OR intrarater OR intra-rater OR intratester OR intra-tester OR interobserver OR inter-observer OR intraobserver OR intra-observer OR intertechnician OR inter-technician OR intratechnician OR intra-technician OR interexaminer OR inter-examiner OR intraexaminer OR intra-examiner OR intraclass correlation OR standard error of measurement OR sensitiv* OR responsive* OR minimal detectable concentration OR interpretab* OR small detectable change OR ceiling effect OR floor effect

### Data management

Endnote software version X9 (Clarivate Analytics) was used to manage citations and bibliographies and store articles found and eliminate duplicates.

#### Study selection

AA carried out the initial search of the databases, after that, two researchers (AA, SA) independently carried out the screening of potentially eligible studies. The screening and selection were carried out in two steps. Step 1: Abstracts and titles using the eligibility criteria. Step 2: Retrieve full text of potentially relevant studies to be screened. Studies were included if both reviewers had agreed on inclusion after screening the full text. In case of any disagreement, a third reviewer (DF) was consulted.

#### Data extraction and data items

Two researchers (AA, SA) independently carried out the data extraction from the included studies. Extracted data items were characteristics of the studies (study design and sample size), characteristics of the participants (age, gender, population), testing instrument, testing protocols, measurement properties (reliability, measurement error, validity, and responsiveness), and results. In case of any disagreement, a third reviewer (DF) was consulted.

#### Risk of bias assessment

Included studies were independently assessed by two reviewers (AA, SA) using the COSMIN RoB checklist for reliability and measurement error of outcome measurement instruments to assess RoB of reliability, measurement error, and criterion validity [14]. The COSMIN RoB checklist for PROMs was used to assess construct and discriminative validity [16]. Both checklists have four scores (very good, adequate, doubtful, and inadequate) [16] that assess measurement properties with regard to design and statistical methods. In case of any disagreement, a third reviewer (DF) was consulted.

#### Data synthesis

Data synthesis of the results was undertaken in accordance with COSMIN guidelines [13]. After assessing the risk of bias, each study was rated using the updated criteria for good measurement properties as sufficient ( +), insufficient (-), or indeterminate (?) [13], then, the overall results of each measurement property per outcome measure per population were rated against the criteria of a good measurement property as sufficient ( +), insufficient (-), inconsistent ( ±), or indeterminate (?) [13]. Table 2 presents the updated criteria for good measurement properties.

**Table 2:** The updated criteria for good measurement properties [13, 18]

Measurement property	Rating	Criteria
Reliability	Sufficient ( +)	ICC or weighted Kappa ≥ 0.7
	Indeterminate (?)	ICC or weighted Kappa not reported
	Insufficient (-)	ICC or weighted Kappa < 0.70
Measurement error	Sufficient ( +)	SDC or LoA < MIC
	Indeterminate (?)	MIC not defined
	Insufficient (-)	SDC or LoA > MIC
Hypothesis testing for construct validity	Sufficient ( +)	The result is in accordance with the hypothesis
	Indeterminate (?)	No hypothesis defined (by the review team)
	Insufficient (-)	The result is not in accordance with the hypothesis
Criterion validity	Sufficient ( +)	Correlation with gold standard ≥ 0.70 OR AUC ≥ 0.70
	Indeterminate (?)	Not all information for ‘ + ’ reported
	Insufficient (-)	Correlation with gold standard < 0.70 OR AUC < 0.70
Responsiveness	Sufficient ( +)	The result is in accordance with the hypothesis OR AUC ≥ 0.70
	Indeterminate (?)	No hypothesis defined (by the review team)
	Insufficient (-)	The result is not in accordance with the hypothesis7 OR AUC < 0.70

*ICC* intraclass correlation coefficient, *SDC* smallest detectable change, *LoA* limits of agreement, *MIC* minimal important change, *AUC* area under curve

The overall level of evidence for each outcome measure and its respective measurement property was then determined independently by two reviewers (AA, SA) using a modified Grading of Recommendations Assessment, Development, and Evaluation (GRADE) approach [19]. Table 3 presents the modified GRADE approach used to rate the overall quality of the evidence. More information on how to downgrade the level of evidence can be found in the COSMIN user manual [19].

**Table 3:** Modified GRADE approach used to rate the overall level of evidence [13]

**Quality of evidence**	**Lower if there is**
High Moderate Low Very low	**Risk of bias** -1 Serious -2 Very serious -3 Extremely serious **Inconsistency** -1 Serious -2 Very serious **Imprecision** -1 Sample size (n = 50–100) -2 Sample size (*n* < 50) **Indirectness** -1 Serious -2 Very serious

## Results

**Fig. 1:**
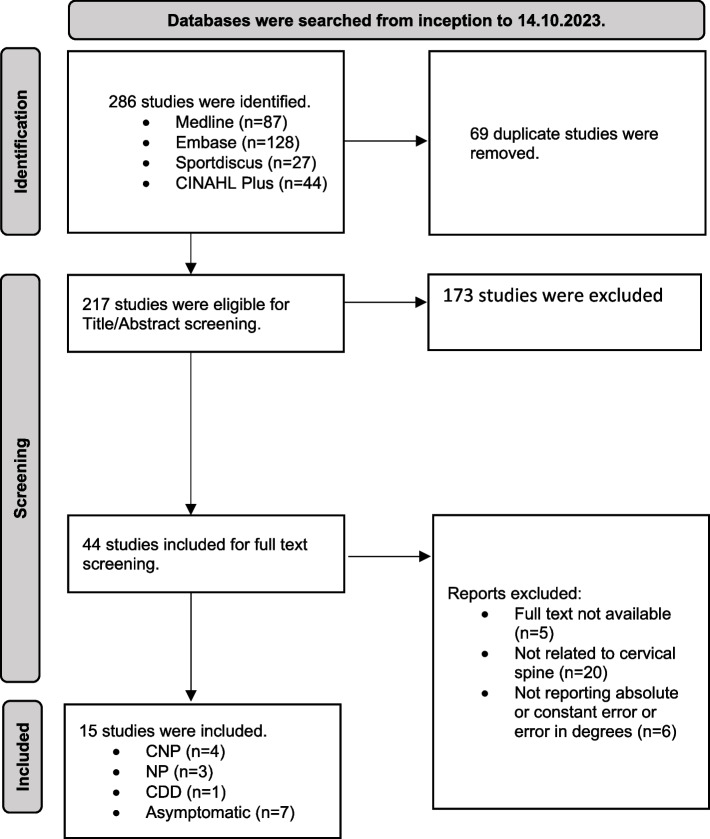
Prisma flow diagram of the study selection process [20]. CNP = chronic neck pain. NP = neck pain. CDD = cervicogenic disc disease. *n* = number of studies

Fifteen studies were included four with CNP, three that did not specify the type of neck pain, one with cervicogenic disc disease, and seven studies that included participants without neck pain. There was a 100% agreement between raters (AA, SA) for the included studies. Search results are summarised in Fig. 1 and Table 4 summarises the extracted data from the included studies.

**Table 4:** Data extracted from the studies included in this review

**Authors**	**Population and sample size**	**Age (mean or range)**	**Testing instrument**	**Testing position**	**Testing procedure**	**Property domain**	**Statistical test used**	**Results**
Artz et al. [21]	Asymptomatic subjects: *n* = 40, (within day *n* = 21, between day *n* = 19)	29.9 years	3-Space Fastrak	Sitting Standing	**Trials:** 3 trials **Movements tested:** **THP:** 25%, 50%, 75% in flexion (randomised)	Reliability within-day and between-day intra-rater (at least 1 week apart)	ICC, SEM	**JPE:** **Sitting:** **Within-day** (ICC range: -0.81–0.66) **Between-day** (ICC range: -0.48–0.77) **SEM range(sitting):** **Within-day** (0.71–1.48) **Between-day** (0.72–0.99) **Standing:** **Within-day** (ICC range: -0.11–0.68) **Between-day** (ICC range: 0.09–0.58) **SEM range (standing):** **Within-day** (0.91–1.48) **Between-day** (0.82–1.22)
Kristjansson et al. [22]	Asymptomatic Subjects: *n* = 19 (12 females)	31.5 years	3-Space Fastrak	Siting	**Trials:** 3 trials each direction or test **Movements tested:** **JPE:** Right rotation, left rotation	Reliability (Intra-rater between-day in the same week)	ICC (2,1)	**Reliability:** **JPE:** **NHP** (ICC range: 0.35–0.44) **THP** (ICC range: 0.69–0.74) **Preset trunk rotation** (ICC range: 0.52–0.74) **Figure of 8 relocation test** (ICC: 0.67)
Lee et al. [23]	Asymptomatic Subjects: *n* = 20 (11 men)	21.9 years	Motion analysis system, CMS 70P	Sitting	**Trials:** 3 trials each direction **Movements tested:** Flexion/extension, left/right rotation, left/right side-bending (not randomised)	Reliability (Intra-rater within day 10 min in between)	ICC (3, 1) SEM	**ICC range**: **NHP** (0.38–0.84) **THP** (-0.48–0.83) **SEM**: 0.3–4
Strimpakos et al. [24]	Asymptomatic Subjects: *n* = 35 (17 males)	18–63 years	Zebris CMS20	Intra-rater: sitting, standing) Inter-rater (standing)	**Trials:** 3 trials **Movements tested:** Flexion, right and left rotation, right and left side bending (not randomised)	Reliability (Intra-rater between day 3 occasion 1 week apart) and Inter-rater reliability, 15 min between assessors (10 subjects only)	ICC (1,1), SEM, Bland and Altman	**Intra-rater** **Sitting**: (ICC range: -0.01–0.35) **Standing**: (ICC range: 0.17–0.5) **SEM**: **Standing** (1.5–3) **Sitting** (1.5–3.5) **Inter-rater** **ICC range**: -0.2–0.64 **SEM**: 0.7–2.9
Pinsault et al. [25]	Asymptomatic subjects: *n* = 44 (22 women)	21.7 years	Laser pointer	Sitting	**Trials:** 10 trials **Movements tested:** Right and left rotation	Reliability (intra-rater 1 h apart)	ICC (2, 1), SEM, LoA	**ICC range:** (0.52–0.81) **SEM** (0.9) **LoA** (-2–2.2)
Kramer et al. [26]	Asymptomatic subjects: *n* = 57 (30 male, 27 females)	18–64 years	Virtual 3D scene via head mounted display + 3-Space Fastrak	Sitting	**Trials:** 8 trials in total **Movements tested:** Flexion, Extension, right and left rotation	Reliability (intrasession and intersession)	ICC (3, 1)	**Intrasession** ICC: 0.63 **Intersession:** ICC: 0.48
Roren et al. [27]	Asymptomatic and CNP subjects: *n* = 82 (41 each group)	Healthy: 30.5 CNP: 54.7 years	Revel visual technique US technique	Sitting	**Trials:** 10 trials each device (5 trials each movement) **Movements tested:** Right and left rotation	Reliability: Intra-rater within day 1 h apart for both devices Criterion validity (Revel visual technique vs US technique) Discriminative validity (healthy vs NP group)	Reliability: ICC, Bland and Altman agreement. Criterion validity: Pearson’s correlation. Discriminative validity: Kappa agreement	**ICC**: **Revel visual system** (0.68) **US technique** (0.62) **Bland and Altman agreement**: **Revel technique** (-3.6–4.2) **US technique** (-3.8–5.6) **Pearson’s correlation range**: 0.946–0.952 **Kappa agreement**: 0.65
Chen and Treleavan [28]	CNP subjects: *n* = 25 Asymptomatic subjects: *n* = 26	18–60 years	Fastrak, Laser pointer	Sitting	**Trials:** 6 trials each movement **Movements tested:** Right and left rotation	Criterion validity Discriminative validity Convergent validity	Pearson's correlation (criterion validity) Spearman's correlation (convergent validity) MANOVA (discriminative validity)	**Discriminative validity**: **Conventional Fastrak** (*p* = 0.28) **Conventional Laser** (*p* = 0.04) **Torsion Fastrak** (*p* = 0.00) **Torsion Laser** (*p* = 0.02) **Enbloc Fastrak (head)** (*p* = 0.43) **Enbloc Fastrak (trunk)** (*p* = 0.42) **Criterion validity:** **Conventional JPE** (*r* = 0.87) **Torsion JPE** (*r* = 0.67) **Convergent validity:** No correlation except Conventional Fastrak (*r* = 0.51)
Wibault et al. [5]	CDD subjects: *n* = 24 Asymptomatic subjects: *n* = 12	CDD: 51 years Healthy: 42 years	Reliability: CROM device Validity: CROM device vs laser pointer	Sitting	**Trials:** Reliability: 3 trials each direction Validity: 8 trials each direction **Movements tested:** Reliability and validity: right and left rotation	Reliability: 24 subjects with CDD (Intra-rater within day 1 h in between) Criterion validity: 12 healthy subjects	ICC (2,1) SEM	**Reliability**: **ICC range**: 0.79–0.85 **SEM range**: 1.4–2 **Validity**: **ICC range**: 0.43–0.91
Dugailly et al. [29]	Asymptomatic and CNP subjects Validity group (*n* = 17) Reliability group (*n* = 5)	42 years	Laser + electrogoniometer	Sitting	**Trials:** 6 trials in each direction **Movements tested:** Right and left rotation, flexion, extension (no evidence of randomisation)	Criterion validity Intra-rater reliability (1 week apart) Convergent validity	Spearman's correlation ICC Bland Altman (LoA)	**ICC range:** 90 cm low speed: 0.22–0.47 90 cm high speed: 0.58–0.79 180 cm low speed: 0.52–0.75 180 cm high speed: 0.8–0.86 **Convergent validity:** JPE vs disability (*r* = 0.32) JPE vs pain intensity (*r* = 0.03), JPE vs pain duration (*r* = 0.14) LoA range: -9 to 9
Burke et al. [30]	Asymptomatic and NP subjects: *n* = 50	NA	CROM, AL	Sitting	**Trials:** 3 trials in each direction for two devices (no randomisation) **Movements tested:** Right and left rotation	Reliability (intra and inter-rater reliability)	ICC (type C)	**ICC range:** Intra-rater CROM: 0.253–0.386 Intra-rater AL: 0.488–0.556 Inter-rater CROM: 0.717–0.773 Inter-rater AL: 0.589–0.75
Alahmari et al. [3]	NP and Asymptomatic subjects: NP: *n* = 36 Asymptomatic: *n* = 33	Healthy: 56 year NP: 36 years	Digital inclinometer	NHP (sittin) THP (sitting and supine)	**Trials:** 3 trials in all tests and directions **Movements tested:** NHP (extension) THP (50% of ROM in flexion, extension, right and left side bending, right and left rotation in a randomised order)	Intra-rater reliability, Inter-rater reliability (≤ 3 working days apart)	ICC (2.1.A) SEM	**Intra-rater reliability:** NHP (ICC range: 0.74–0.78) (SEM: 1.78–1.88) THP: (ICC range: 0.7–0.83) (SEM: 1.78–1.88) **Inter-rater reliability:** NHP (ICC range: 0.74–0.79) (SEM 1.79–1.87) THP (ICC range: 0.62–0.84) (SEM: 1.5–2.23)
Goncalves and Silva [31]	Asymptomatic vs CNP subjects: *n* = 66 (33 each group)	Healthy: 43.6 years NP 43.5 years	Laser pointer on a helmet	Sitting	**Trials:** 6 trials each direction **Movements tested:** Right and left rotation	Reliability (Intra-rater within day and between day with 1–2 days in between) Construct validity	Reliability: ICC (2,1) SEM Validity: t-tests or Mann Whitney, Pearson correlation coefficient, Spearman's correlation coefficient	**Chronic Neck pain:** **Within day ICC range:** HRNT: 0.9–0.93 TT: 0.88–0.9 HR30T: 0.73–0.79 F8T: 0.89–0.93 **Between day ICC range:** HRNT: 0.61–0.85 TT: 0.58–0.71 HR30T: 0.67–0.7 F8T: 0.66–0.85 **Asymptomatic:** **Within day ICC range:** HRNT: 0.79–0.89 TT: 0.75–0.87 HR30T: 0.78–0.83 F8T: 0.83–0.93 **Between day ICC range:** HRNT: 0.75–0.8 TT: 0.57–0.59 HR30T: 0.55–0.76 F8T: 0.8–0.83 **All tests between groups (NP vs healthy) were < 0.05 but the HR30T** **Between test correlations ranged between 0.35 and 0.61 and correlations between proprioceptive tests and catastrophizing, fear of movement and disability were, in general, lower than 0.3**
Nikkhoo et al. [32]	Asymptomatic (35 participants)	21.2	US MOCAP (CMS 10, Zebris) + IMU-based mobile devices	Sitting	**Trials:** 5 trials **Movements tested:** Flexion, extension, right and left rotation	Within-day and between-day intra-rater reliability (5–7 days in between) Measurement error Criterion validity	**Reliability:** ICC two-way mixed model, **Measurement error:** SEM **Validity:** Pearson’s correlation	**Reliability:** **Within-day (US MOCAP**): ICC range 0.83–0.93 **Between-day (US MOCAP**): ICC range 0.69–0.85 **Within-day (IMU**): ICC range 0.66–0.91 **Between-day (IMU**): ICC range 0.63–0.76 **Validity:** **r range:** 0.74–0.83
Cid Et al. [33]	Asymptomatic (14 men, 14 women) NP (13 women)	24.4, 23.1, 26.6	Laser pointer	Sitting	**Trials:** 10 trials **Movements tested:** Right and left rotation	Within-day intra-rater reliability (at least 7 days in between)	**Reliability:** ICC (two-way mixed model)	**Reliability (NHP):** **NP:** ICC range 0.77–0.86 **Asymptomatic:** ICC range -0.16–0.5

*CNP* chronic neck pain, *NP* neck pain, *n* number of subjects, *NHP* neutral head position, *THP* target head position, *Wk* weighted kappa, *ICC* intraclass correlation coefficient, *SEM* standard error of measurement, *LoA* limits of agreement, *JPE* joint position error, *CROM* cervical range of motion, *HRNT* head repositioning to neutral, *TT* torsion test, *F8T* figure-of-eight test, *HR30T* head repositioning to 30 degrees test, *CDD* cervical disc disease, *IMU* inertial measurement unit, *r* Pearson’s or Spearman’s correlation

### Absolute joint position error for people with neck pain

#### Intra-rater reliability

For the NHP test, six studies investigated intra-rater reliability of absolute JPE. One study included participants with CDD [5] testing right and left rotation using a CROM device and 3 trials for their assessment in sitting position, however only the NHP test was reported. This study was rated as inadequate in the RoB checklist and sufficient in the updated criteria for good measurement properties. Three studies mentioned neck pain participants but failed to report type or duration of neck pain [3, 30, 33]. Alahmari et al. [3] carried out their intra-rater reliability assessment for the NHP test, it was rated as inadequate in the RoB checklist and sufficient in the updated criteria for good measurement properties. Burke et al. [30] carried out their intra-rater reliability using two devices, the CROM and laser. Both were rated as inadequate in the RoB checklist and insufficient in the updated criteria for good measurement properties. Cid et al. [33] investigated the intra-rater reliability of the NHP, it was rated as inadequate in the RoB checklist and sufficient in the updated criteria for good measurement properties. Moreover, two studies included CNP participants [27, 31], and tested both right and left rotation in sitting position. Roren et al. [27] included 5 trials in their assessment, and used a laser pointer and US device. Both parts were rated as inadequate in the RoB checklist and insufficient in the updated criteria for good measurement properties. Goncalves and Silva [31] carried out within-day and between-day intra-rater reliability investigations of different types of NHP tests: NHP, figure of 8 (F8T) relocation test, and torsion test (TT). All investigations for were rated as doubtful in RoB checklist and sufficient in the updated criteria for good measurement properties. Nine studies showed sufficient results and four studies showed insufficient results. Therefore, the overall rating was taken. The overall rating of the intra-rater reliability was rated as sufficient, but the quality of evidence was downgraded to very low due to inconsistency of results and risk of bias (multiple studies with doubtful/inadequate ratings and inconsistency of results) (Table 5).

**Table 5:** Summary of measurement properties of the measure of absolute JPE

**Neutral head position (Neck Pain population)**	**Summary or pooled results**	**Overall rating**	**Quality of evidence**
Intra-rater reliability	ICC: 0.58–0.93 Total sample size: 580	Sufficient	Very low evidence for sufficient intra-rater reliability • Nine studies showed sufficient results, 4 showed insufficient results (Inconsistent results) • Multiple studies with doubtful/inadequate rating (risk of bias) • No imprecision • No indirectness
Inter-rater reliability	ICC: 0.58–0.79 Total sample size: 169	Sufficient	Low evidence for sufficient inter-rater reliability • Three studies showed sufficient results • Multiple studies with inadequate rating • No inconsistency • No imprecision • No indirectness
Measurement error	Total sample size: 736	Indeterminate	Not possible to apply GRADE as the minimal important change was not provided
Convergent validity	Correlation (*r* < 0.5) Total sample size: 1890	Insufficient	Low evidence for insufficient convergent validity • Thirteen studies showed sufficient results, 17 studies showed insufficient results (Inconsistent results) • Multiple studies with adequate rating (no risk of bias) • No indirectness • No imprecision
Discriminative validity	Total sample size: 496	Indeterminate	Very Low evidence for indeterminate discriminative validity • Seven studies were indeterminate and 1 study was sufficient (inconsistent results) • Multiple studies with inadequate rating • No imprecision • No indirectness
Criterion validity	*r* = 0.87–0.95 Total sample size: 184	Sufficient	Low evidence for sufficient criterion validity • Two studies were sufficient, 1 was insufficient (inconsistent results) • Multiple studies with adequate rating (no risk of bias) • No imprecision • No indirectness
**Target head position (Neck Pain population)**	**Summary of pooled results**	**Overall rating**	**Quality of evidence**
Intra-rater reliability	ICC: 0.67–0.83 Total sample size: 135	Sufficient	Low evidence for sufficient intra-rater reliability • Three studies showed sufficient results • Multiple studies with doubtful/inadequate rating • No imprecision • No indirectness
Inter-rater reliability	ICC: 0.58–0.84 Total sample size: 69	Sufficient	Very low evidence of sufficient inter-rater reliability • One study showed sufficient results • One study with inadequate rating (risk of bias) • Imprecision • No indirectness
Measurement error	Total sample size: 204	Indeterminate	Not possible to apply GRADE as the minimal important change was not provided
**Neutral head position (asymptomatic population)**	**Summary or pooled results**	**Overall rating**	**Quality of evidence**
Intra-rater reliability	ICC: 0.52–0.93 Total sample size: 537	Sufficient	Very low evidence of sufficient intra-rater reliability • Eleven studies showed sufficient results, 6 showed insufficient results (inconsistent results) • Multiple studies with doubtful/inadequate rating (risk of bias) • No imprecision • No indirectness
Inter-rater reliability	ICC: -0.2–0.64 Total sample size: 35	Insufficient	Very low evidence of insufficient inter-rater reliability • One study showed insufficient results • One study with inadequate rating (risk of bias) • Imprecision (sample size < 100) • No inconsistency • No indirectness
Measurement error	Total sample size: 509	Indeterminate	Not possible to apply GRADE as the minimal important change was not provided
Intra-session reliability	ICC: 0.63 Total sample size: 57	Insufficient	Very low evidence of insufficient intra-session reliability • One study with doubtful rating (risk of bias) • Imprecision • No inconsistency • No indirectness
Inter-session reliability	ICC: 0.48 Total sample size: 57	Insufficient	Very low evidence of insufficient intra-session reliability • One study with doubtful rating (risk of bias) • Imprecision • No inconsistency • No indirectness
Criterion validity	Total sample size: 71	Inconsistent	Not possible to apply GRADE due to inconsistency of results • One study showed indeterminate results and one showed sufficient results
Target head position (asymptomatic population)	Summary of pooled results	Overall rating	Quality of evidence
Intra-rater reliability	ICC: -0.48–0.83 Total sample size: 165	Sufficient	Very low evidence of sufficient intra-rater reliability • Four studies showed sufficient results, three showed insufficient results (inconsistency of results) • Multiple studies with doubtful/inadequate rating (risk of bias) • No indirectness • No imprecision
Measurement error	Total sample size: 165	Indeterminate	Not possible to apply GRADE as the minimal important change was not provided

For the THP test, two studies tested the intra-rater reliability of the THP test [3, 31]. Alahmari et al. [3] was rated as inadequate in the RoB checklist and sufficient in the updated criteria for good measurement properties. Goncalves and Silva [31] carried out a within-day and between-day testing. Both investigations were rated as doubtful in the RoB checklist and sufficient in the updated criteria for good measurement properties. The overall rating of the intra-rater reliability of the THP test was rated as sufficient, but the quality of evidence was downgraded to low due to risk of bias (multiple studies with doubtful/inadequate rating) (Table 5).

#### Inter-rater reliability

Only two studies investigated inter-rater reliability of the NHP test in this population, and both did not report type of neck pain. Alahmari et al. [3] was rated as inadequate in the RoB checklist and sufficient in the updated criteria for good measurement properties. Burke et al. [30] carried out their investigation using two devices the laser pointer and the CROM. Both were rated as inadequate in the RoB checklist and sufficient in the updated criteria for good measurement properties. A total of three investigation showing sufficient results. The overall rating was rated as sufficient, but the quality of evidence was downgraded to low due to risk of bias (multiple studies with inadequate ratings) (Table 5).

#### Measurement error

For the THP test, five studies investigated measurement error [3, 5, 27, 30, 31]. GRADE was not possible to apply due to minimal important change (MIC) not provided (Table 5). For the THP test, two studies investigated measurement error [3, 31]. GRADE was not possible to apply as the minimal important change was not provided (Table 5).

#### Convergent validity

Two studies investigated the convergent validity in this population and were on CNP people. Chen and Treleaven [28] correlated three JPE tests (conventional, TT, Enbloc) with the neck disability index (NDI) and the visual analogue scale (VAS). All parts were rated as adequate in the RoB checklist and insufficient in the updated criteria for good measurement properties, apart from the correlation of JPE conventional with VAS, which showed sufficient results. Goncalves and Silva [31] correlated four JPE tests (NHP, THP, TT, and F8T) against each other and against disability, pain catastrophising, and fear of movement questionnaires. All parts were rated as adequate in the RoB checklist. Correlation of the tests against the questionnaires were rated as insufficient in the updated criteria for good measurement properties, while correlation of tests against each other were rated as sufficient. Seventeen investigations showed insufficient results and thirteen studies showed sufficient results. The overall rating was taken and rated as insufficient, and the quality of evidence was downgraded to low due to inconsistency of results (Table 5).

#### Discriminative validity

Three studies investigated the discriminative validity in people with CNP. Chen and Treleaven [28] used three tests (JPE conventional, TT, Enbloc), Goncalves and Silva [31] used four tests (NHP, THP, TT, F8T), and Roren et al. [27] used the NHP test. All investigation were rated as inadequate in the RoB checklist. All studies were rate as indeterminate in the updated criteria for good measurement properties due to improper statistical tests used for analysis, apart from the study by Roren et al. [27], which was rated as sufficient. Seven studies showed indeterminate results and one study showed sufficient results. The overall rating of the discriminative validity was rated as indeterminate, and the quality of evidence was downgraded to very low due to inconsistency of results and risk of bias (multiple studies with inadequate rating) (Table 5).

#### Criterion validity

The criterion validity was reported only in CNP population testing for only right and left rotation. Roren et al. [27] correlated the laser pointer against an US device in sitting position for the NHP test only. This study was rated as inadequate in the RoB checklist and sufficient in the updated criteria for good measurement properties. Chen and Treleavan [28] correlated the laser pointer against the 3-Space Fastrak for both the NHP and TT in sitting position. Both parts were rated as adequate in the RoB checklist. The conventional JPE was rated as sufficient, and the TT was rated as insufficient in the updated criteria for good measurement properties. Two investigations showed sufficient results and one showed insufficient results. The overall rating was rated as sufficient, and the quality of evidence was downgraded to low due to inconsistency of results (Table 5).

### Absolute joint position error for asymptomatic people

#### Intra-rater reliability

A total of six studies investigated intra-rater reliability of the NHP test in this population. Kristjansson et al. [22] carried their investigation on four JPE tests: NHP, Preset trunk rotation, and F8T relocation test. All parts were rated as inadequate in the RoB checklist. The NHP and F8T investigations were rated as insufficient, and Present trunk rotation investigation was rated as sufficient in the updated criteria for good measurement properties. Strimpakos et al. [24] carried out their intra-rater investigation in sitting and standing. Both were rated as inadequate in the RoB checklist and insufficient in the updated criteria for good measurement properties. Pinsault et al. [25] was rated as doubtful in the RoB checklist and sufficient in the updated criteria for good measurement properties. Goncalves and Silva [31] carried out within-day and between day investigations for three NHP tests (NHP, TT, and F8T). All investigations were rater as doubtful in the RoB checklist. The between-day investigation of the TT was rated as insufficient, while the remaining investigations were rated as sufficient in the updated criteria for good measurement properties. Nikkhoo et al. [34] carried out within-day and between-day investigations using US MOCAP and IMU devices. All investigations were rated as doubtful in the RoB checklist and sufficient in the updated criteria for good measurement properties. Cid et al. [33] was rated as doubtful in the RoB checklist and insufficient in the updated criteria for good measurement properties. Eleven studies showed sufficient results and six studies showed insufficient results. The overall rating was sufficient, and the quality of evidence was downgraded to very low due to inconsistency of results and risk of bias (multiple studies with doubtful/inadequate rating) (Table 5).

Regarding the THP test, three studies investigated the intra-rater reliability of this test in this population [21, 22, 31] Artz et al. [21] carried out within-day and between-day intra-rater reliability of THP test only in sitting and standing. All parts were rated as inadequate in the RoB checklist and insufficient in the updated criteria for good measurement properties, apart from the between-day assessment in sitting, which was rated as sufficient. Kristjansson et al. [22] was rated as inadequate in the RoB checklist and sufficient in the updated criteria for good measurement properties. Goncalves and Silva [31] carried out a within-day and between-day investigations, both investigation were rated as doubtful in the RoB checklist and sufficient in the updated criteria for good measurement properties. Four studies showed sufficient results and three studies showed insufficient results. The overall rating was rated as sufficient, but the quality of evidence was downgraded to very low due to risk of bias and inconsistency of results (Table 5).

#### Inter-rater reliability

Only one study investigated inter-rater reliability of the NHP test [24] in this population. This study was rated as inadequate in the RoB checklist and insufficient in the updated criteria for good measurement properties. The overall rating was insufficient, and the quality of evidence was downgraded to very low due to risk of bias and low imprecision (sample size < 100) (Table 5).

#### Intra-session reliability

Only one study [26] investigated in intra-session reliability of the NHP test in this population. This study was rated as doubtful in the RoB checklist and insufficient in the updated criteria for good measurement properties. The overall rating was insufficient, and the quality of evidence was very low due to risk of bias and imprecision (sample size < 100) (Table 5).

#### Inter-session reliability

Only one study [26] investigated in inter-session reliability of the NHP test in this population. This study was rated as doubtful in the RoB checklist and insufficient in the updated criteria for good measurement properties. The overall rating was insufficient, and the quality of evidence was very low due to risk of bias and imprecision (sample size < 100) (Table 5).

#### Measurement error

For the NHP test, six studies investigated measurement error [21, 22, 24, 25, 31, 34]. GRADE was not possible to apply due to MIC no provided. For the THP test, three studies investigated measurement error [21, 22, 31]. GRADE was not possible to apply as the minimal important change was not provided.

#### Criterion validity

Two studies investigated criterion validity in this population. Wibault et al. [5] was rated as doubtful in the RoB checklist and indeterminate in the updated criteria for good measurement properties. Nikkhoo et al. [32] was rated as adequate in the RoB checklist and sufficient in the updated criteria for good measurement properties. We were not able to take an overall rating as one study showed sufficient results and the other one showed indeterminate results. Therefore, the overall rating was indeterminate, and no GRADE was applied due to inconsistency of results (Table 5).

### Constant joint position error for asymptomatic people

#### Intra-rater reliability

Two studies investigated the intra-rater reliability of the NHP test. Lee et al. [23] was rated as inadequate in the RoB checklist and sufficient in the updated criteria for good measurement properties. Dugailly et al. [29] carried out four intra-rater reliability investigation of the NHP test; low and fast speeds at 90cm and 180cm from a target. All four parts were rated as inadequate in the RoB checklist. Only the low speed at 90cm was rated as insufficient, while the remaining three were rated as sufficient in the updated criteria for good measurement properties. Four studies showed sufficient result, one study showed insufficient results. The overall rating was sufficient, and the quality of evidence was downgraded to very low due to inconsistency of results, risk of bias (multiple studies with inadequate ratings), and imprecision (sample size < 100) (Table 6).

**Table 6:** Summary of measurement properties of the measure of constant JPE

Neutral head position (asymptomatic population)	Summary or pooled results	Overall rating	Quality of evidence
Intra-rater reliability	ICC: 0.38–0.86 Total sample size: 40	Sufficient	Very low evidence of sufficient intra-rater reliability • Four studies showed sufficient results and 1 showed insufficient results (inconsistency) • Multiple studies with inadequate rating (risk of bias) • Imprecision (sample size < 100) • No indirectness
Measurement error	Total sample size: 40	Indeterminate	Not possible to apply GRADE as the minimal important change was not provided
Convergent validity	*r* = 0.03–0.32 Total sample size: 213	Insufficient	High evidence for insufficient convergent validity • Multiple studies with adequate rating (no risk of bias) • No inconsistency of results • No imprecision • No indirectness
Criterion validity	Total sample size: 17	Indeterminate	Very Low evidence for indeterminate criterion validity • One study with doubtful rating (risk of bias) • Imprecision (sample size < 50) • No indirectness
Target head position (asymptomatic population)	Summary of pooled results	Overall rating	Quality of evidence
Intra-rater reliability	ICC: -0.47–0.83 Total sample size: 20	Sufficient	Very low quality of evidence for sufficient intra-rater reliability • One study showed sufficient results • One study with inadequate rating (risk of bias) • Imprecision • No inconsistency • No indirectness
Measurement error	Total sample size: 20	Indeterminate	Not possible to apply GRADE as the minimal important change was not provided

For the THP test, only one study investigated the intra-rater reliability of this test [23]. This study was rated as inadequate in the RoB checklist and sufficient in the updated criteria for good measurement properties. The overall rating was sufficient, but the quality of evidence was downgraded to very low due to risk of bias and imprecision (sample size < 100) (Table 6).

#### Measurement error

For the NHP test, two studies investigated the measurement error in this population [23, 29]. GRADE was not possible to apply due to MIC not provided (Table 6). For the THP, only one study investigated measurement error [23]. GRADE was not possible to apply as the minimal important change was not provided (Table 6).

#### Convergent validity

One study by Dugailly et al. [29] correlated the JPE test against disability questionnaire, pain duration, and pain intensity. All parts were rated as adequate in the RoB checklist and insufficient in the updated criteria for good measurement properties. The overall rating was insufficient, and the quality of evidence was high due to multiple studies with adequate ratings (no risk of bias) (Table 6).

#### Criterion validity

The criterion validity was reported only once by Dugailly et al. [29]. This study was rated as doubtful in the RoB checklist and indeterminate in the updated criteria for good measurement properties. The overall rating was indeterminate, and the quality of evidence was downgraded to very low due to risk of bias and imprecision (sample size < 50) (Table 6).

## Discussion

This is the first systematic review to synthesise and appraise the measurement properties of cervical JPE in people with and without neck pain using the COSMIN checklist. Our search yielded 8 studies that included neck pain participants and 7 in which asymptomatic participants were included. Absolute and constant errors were reported in this review since they are recommended when assessing JPE [35]. The large range of testing procedures used in the studies reviewed highlight the lack of any consensus in the literature on how best to assess JPE. A key factor contributing to this may be the heterogeneity of neck pain participants recruited for the reviewed studies, each with different clinical features. Given these differences in testing procedures and the vast range in types of neck pain, it is difficult to draw any general conclusions on the gold standard for testing the measurement properties of cervical JPE.

Similar to other systematic reviews, the current systematic review highlighted several issues with the quality of the included studies [36, 37]. Most of the included studies in this review were rated as inadequate or doubtful in the RoB checklist with an overall quality of the evidence being low to very low, apart from the convergent validity of the constant JPE, which was high. This was due to a failure in adhering to COSMIN guidelines when carrying out investigations of measurement properties of outcome measures. For example, according to COSMIN, the time-interval should be long enough to prevent recall bias, and short enough to ensure that the patients have not been changed on the construct to be measured [13]. When assessing the RoB for reliability and measurement error, there are no guidelines for the time-interval between sessions, therefore, this section was rated as doubtful. Other issues highlighted were statistical tests used for validity investigations. COSMIN recommends Pearson’s or Spearman’s correlation for validity assessment, which the criterion validity in the constant JPE did not use. Therefore, some of the included studies were rated as indeterminate in the updated criteria for good measurement properties. A further limitation in the included studies was when the model of the ICC used for reliability assessment was not stated. When using the RoB checklist for reliability and measurement error [14], if a study used ICC and reported the model used, it should be rated as very good; if the study used ICC but failed to report the model, then it should be rated as inadequate. Three studies failed to report the ICC model used [21, 27, 29], thus, they were rated as inadequate in the RoB checklist. Reporting the ICC model is important because the model used and the type of coefficient will impact on the magnitude of the ICC [38]. Failure to report the ICC model will affect the study’s generalisability and interpretation of the results. Inclusion of a replicable measure of response stability will aid the interpretation of results and comparison between studies.

Another issue in the current review was the inconsistency of results for the criterion validity of absolute JPE in the asymptomatic population. This inconsistency was probably due to differences in statistical tests used for validity assessment and variations in testing protocols. For example, Wibault et al. [5] correlated the CROM device against a laser pointer after returning from right and left rotation using three trials per movement in their assessment. They used the ICC for their validity assessment, which is not recommended by COSMIN, and thus were rated as indeterminate in the updated criteria for good measurement properties. Nikkhoo et al. [32] correlated the US MOCAP against IMUs after returning from flexion, extension, and bilateral rotation using five trials per movement. This study was rated as sufficient in the updated criteria for good measurement properties. Therefore, it was not possible to draw an overall rating for this measurement property due to inconsistency of the results and it was rated as indeterminate. The convergent validity on the other hand was rated as high. This was due to no risk of bias in the included studies; however, it did not show sufficient results. Sample size was another issue that affected the overall rating of an outcome measure. When applying the modified GRADE approach, sample size should be ≥ 100. However, the total sample size of the inter-rater reliability of absolute JPE in the asymptomatic population was 62 participants; this led to downgrading the overall evidence to one level. Similarly, the criterion validity of constant JPE was downgraded to two levels due to sample size < 50. In addition, the wording around reliability studies was challenging as several studies did not report the word ‘’reliability’’ in the title of the study, affecting the quality of the study.

Furthermore, the current systematic review highlighted gaps in the literature when testing the measurement properties of the measure of cervical JPE. First, the testing position. Most of the included studies carried out their investigations in sitting. Only two studies carried out their investigation in sitting as well as standing [21, 24]. However, these two studies did not include any neck pain patients, and only asymptomatic participants were recruited. In addition, they reported only constant JPE, failing to report absolute JPE. A second gap was the lack of investigation of inter-rater reliability of constant JPE in people with neck pain. The third gap we uncovered was regarding the criterion validity of absolute JPE. Although this property was investigated twice, it was limited to right and left rotation. Lastly, the domain of responsiveness was not reported in our systematic review.

### Methodological considerations

This is the first systematic review to summarise and appraise the evidence of measurement properties of the cervical JPE measure using COSMIN guidelines. Two raters carried out the study selection, data extraction, the risk of bias checklist, and the GRADE approach minimising bias, which is considered a strength of this systematic review. Additionally, we included studies that have reported absolute and constant errors, which is recommended when testing cervical proprioception [35]. Prospective registration with PROSPERO is another strength of this review. A potential limitation is that the principle of lowest rating counts when using the COSMIN risk of bias checklist, thus underestimating the overall quality of the study, and potentially downgrading the overall quality of the evidence.

### Recommendations for future research

Additional research is clearly warranted to assess the measurement properties of the measure of JPE in people with and without neck pain. Another recommendation is to report both absolute and constant errors in future research. Also, assessing the measurement properties of the measure of JPE in standing in addition to sitting is recommended, as well as reporting absolute and constant error for both. Responsiveness of the measure of JPE was not investigated, which we recommend investigating in future research.

## Conclusion

Conclusions about the measurement properties of the measure of cervical JPE were difficult to draw due to lack of consensus on testing procedures and tools used. Further high-quality research to overcome the risk of bias in the included studies is required. Studies are also required to investigate the responsiveness of this measure.

### Supplementary Information

**Additional file 1.**

**Additional file 2.**

**Additional file 3.**

**Additional file 4.**

**Additional file 5.**

**Additional file 6.**

## Data Availability

All data generated or analysed during this study are included in this published article and its supplementary information files.
