# Supplementary material for: Measurement properties of cervical joint position error in people with and without neck pain: a systematic review and narrative synthesis
Source: BMC Musculoskelet Disord. 2024 Jan 10;25:44. doi: 10.1186/s12891-023-07111-4 (PMC10777525; doi:10.1186/s12891-023-07111-4)
Supplement: Supplementary file 1 — Additional file 1. [file 12891_2023_7111_MOESM1_ESM.docx]

**Construct validity – Risk of bias**

| *Score: V= very good; A = adequate; D = doubtful; I = inadequate; N= not applicable* | |
| --- | --- |
|  |  |
|  |  |
| ***9. Hypotheses testing for construct validity*** | |
| **9a. Comparison with other outcome measurement instruments (convergent validity)** | |
| 1 | Is it clear what the comparator instrument(s) measure(s)? |
| 2 | Were the measurement properties of the comparator instrument(s) adequate? |
| 3 | Was the statistical method appropriate for the hypotheses to be tested? |
| 4 | Were there any other important flaws? |
|  | **TOTAL** *Lowest score of items 1-4* |

| Goncalves and Silva (HRNT vs Disability) | | | Goncalves and Silva (HRNT vs pain catastrophising) | | | Goncalves and Silva (HRNT vs fear of movement) | | | Goncalves and Silva (TT vs Disability) | | |
| --- | --- | --- | --- | --- | --- | --- | --- | --- | --- | --- | --- |
|  |  |  |  |  |  |  |  |  |  |  |  |
| **rater 1** | **rater 2** | **Consensus** | **rater 1** | **rater 2** | **Consensus** | **rater 1** | **rater 2** | **Consensus** | **rater 1** | **rater 2** | **Consensus** |
| V | V | V | V | V | V | V | V | V | V | V | V |
| V | V | V | V | V | V | V | V | V | V | V | V |
| V | V | V | V | V | V | V | V | V | V | V | V |
| A (sample size) | V | A | A (sample size) | V | A | A (sample size) | V | A | A (sample size) | V | A |
| A | V | A | A | V | A | A | V | A | A | V | A |

| Goncalves and Silva (TT vs pain catastrophising) | | | Goncalves and Silva (TT vs fear of movement) | | | Goncalves and Silva (HR30T vs Disability) | | | Goncalves and Silva (HR30T vs pain catastrophising) | | |
| --- | --- | --- | --- | --- | --- | --- | --- | --- | --- | --- | --- |
|  |  |  |  |  |  |  |  |  |  |  |  |
| **rater 1** | **rater 2** | **Consensus** | **rater 1** | **rater 2** | **Consensus** | **rater 1** | **rater 2** | **Consensus** | **rater 1** | **rater 2** | **Consensus** |
| V | V | V | V | V | V | V | V | V | V | V | V |
| V | V | V | V | V | V | V | V | V | V | V | V |
| V | V | V | V | V | V | V | V | V | V | V | V |
| A (sample size) | V | A | A (sample size) | V | A | A (sample size) | V | A | A (sample size) | V | A |
| A | V | A | A | V | A | A | V | A | A | V | A |

| Goncalves and Silva (HR30 T vs fear of movement) | | | Goncalves and Silva (F8T vs Disability) | | | Goncalves and Silva (F8T vs pain catastrophising) | | | Goncalves and Silva (F8T vs fear of movement) | | |
| --- | --- | --- | --- | --- | --- | --- | --- | --- | --- | --- | --- |
|  |  |  |  |  |  |  |  |  |  |  |  |
| **rater 1** | **rater 2** | **Consensus** | **rater 1** | **rater 2** | **Consensus** | **rater 1** | **rater 2** | **Consensus** | **rater 1** | **rater 2** | **Consensus** |
| V | V | V | V | V | V | V | V | V | V | V | V |
| V | V | V | V | V | V | V | V | V | V | V | V |
| V | V | V | V | V | V | V | V | V | V | V | V |
| A (sample size) | V | A | A (sample size) | V | A | A (sample size) | V | A | A (sample size) | V | A |
| A | V | A | A | V | A | A | V | A | A | V | A |

| Goncalves and Silva (HRNT vs TT) | | | Goncalves and Silva (HRNT vs HR30T) | | | Goncalves and Silva (HRNT vs F8T) | | | Goncalves and Silva (TT vs HRNT) | | |
| --- | --- | --- | --- | --- | --- | --- | --- | --- | --- | --- | --- |
|  |  |  |  |  |  |  |  |  |  |  |  |
| **rater 1** | **rater 2** | **Consensus** | **rater 1** | **rater 2** | **Consensus** | **rater 1** | **rater 2** | **Consensus** | **rater 1** | **rater 2** | **Consensus** |
| V | V | V | V | V | V | V | V | V | V | V | V |
| V | V | V | V | V | V | V | V | V | V | V | V |
| V | V | V | V | V | V | V | V | V | V | V | V |
| A (sample size) | V | A | A (sample size) | V | A | A (sample size) | V | A | A (sample size) | V | A |
| A | V | A | A | V | A | A | V | A | A | V | A |

| Goncalves and Silva (TT vs HR30T) | | | Goncalves and Silva (TT vs F8T) | | | Goncalves and Silva ( HR30T vs HRNT) | | | Goncalves and Silva (HR30T vs TT) | | |
| --- | --- | --- | --- | --- | --- | --- | --- | --- | --- | --- | --- |
|  |  |  |  |  |  |  |  |  |  |  |  |
| **rater 1** | **rater 2** | **Consensus** | **rater 1** | **rater 2** | **Consensus** | **rater 1** | **rater 2** | **Consensus** | **rater 1** | **rater 2** | **Consensus** |
| V | V | V | V | V | V | V | V | V | V | V | V |
| V | V | V | V | V | V | V | V | V | V | V | V |
| V | V | V | V | V | V | V | V | V | V | V | V |
| A (sample size) | V | A | A (sample size) | V | A | A (sample size) | V | A | A (sample size) | V | A |
| A | V | A | A | V | A | A | V | A | A | V | A |

| Goncalves and Silva (HR30T vs F8T) | | | Goncalves and Silva (F8T vs HRNT) | | | Goncalves and Silva (F8T vs TT) | | | Goncalves and Silva (F8T vs HR30T) | | |
| --- | --- | --- | --- | --- | --- | --- | --- | --- | --- | --- | --- |
|  |  |  |  |  |  |  |  |  |  |  |  |
| **rater 1** | **rater 2** | **Consensus** | **rater 1** | **rater 2** | **Consensus** | **rater 1** | **rater 2** | **Consensus** | **rater 1** | **rater 2** | **Consensus** |
| V | V | V | V | V | V | V | V | V | V | V | V |
| V | V | V | V | V | V | V | V | V | V | V | V |
| V | V | V | V | V | V | V | V | V | V | V | V |
| A (sample size) | V | A | A (sample size) | V | A | A (sample size) | V | A | A (sample size) | V | A |
| A | V | A | A | V | A | A | V | A | A | V | A |

| Chen and Treleavan (JPE conventional vs VAS) | | | Chen and Treleavan (JPE Torsion vs VAS) | | | Chen and Treleavan (JPE Enbloc vs VAS) | | | Chen and Treleavan (JPE conventional vs NDI) | | |
| --- | --- | --- | --- | --- | --- | --- | --- | --- | --- | --- | --- |
|  |  |  |  |  |  |  |  |  |  |  |  |
| **rater 1** | **rater 2** | **Consensus** | **rater 1** | **rater 2** | **Consensus** | **rater 1** | **rater 2** | **Consensus** | **rater 1** | **rater 2** | **Consensus** |
| V | V | V | V | V | V | V | V | V | V | V | V |
| V | V | V | V | V | V | V | V | V | V | V | V |
| V | V | V | V | V | V | V | V | V | V | V | V |
| A (sample size) | A (sample size) | A | A (sample size) | A (sample size) | A | A (sample size) | A (sample size) | A | A (sample size) | A (sample size) | A |
| A | A | A | A | A | A | A | A | A | A | A | A |

| Chen and Treleavan (JPE Torsion vs NDI) | | | | Chen and Treleavan (JPE Enbloc vs NDI) | | | Dugailly (JPE vs disability) | | | Dugailly (JPE vs pain intensity) | | | | Dugailly (JPE vs pain duration) | | |
| --- | --- | --- | --- | --- | --- | --- | --- | --- | --- | --- | --- | --- | --- | --- | --- | --- |
|  | |  |  |  |  |  |  |  |  |  | |  |  |  |  |  |
| **rater 1** | | **rater 2** | **Consensus** | **rater 1** | **rater 2** | **Consensus** | **rater 1** | **rater 2** | **Consensus** | **rater 1** | | **rater 2** | **Consensus** | **rater 1** | **rater 2** | **Consensus** |
| V | | V | V | V | V | V | V | V | V | V | | V | V | V | V | V |
| V | | V | V | V | V | V | V | V | V | V | | V | V | V | V | V |
| V | | V | V | V | V | V | V | V | V | V | | V | V | V | V | V |
| A (sample size) | | A (sample size) | A | A (sample size) | A (sample size) | A | A (sample size) | A (sample size) | A (sample size) | A (sample size) | | A (sample size) | A (sample size) | A (sample size) | A (sample size) | A (sample size) |
| A | | A | A | A | A | A | A | A | A | A | | A | A | A | A | A |
|  |  | | | | | | | | | |  |  |  |  |  |  |
| **9b. Comparison between subgroups (discriminative or known-groups validity)** | | | | | | | | | | |  |  |  |  |  |  |
| 5 | Was an adequate description provided of important characteristics of the subgroups? | | | | | | | | | |  |  |  |  |  |  |
| 6 | Was the statistical method appropriate for the hypotheses to be tested? | | | | | | | | | |  |  |  |  |  |  |
| 7 | Were there any other important flaws? | | | | | | | | | |  |  |  |  |  |  |
|  | **TOTAL** *Lowest score of items 5-7* | | | | | | | | | |  |  |  |  |  |  |

| Goncalves and Silva (HRNT) | | | Goncalves and Silva (TT) | | | Goncalves and Silva (HR30T) | | | Goncalves and Silva (F8T) | | |
| --- | --- | --- | --- | --- | --- | --- | --- | --- | --- | --- | --- |
| **rater 1** | **rater 2** | **Consensus** | **rater 1** | **rater 2** | **Consensus** | **rater 1** | **rater 2** | **Consensus** | **rater 1** | **rater 2** | **Consensus** |
| V | V | V | V | V | V | V | V | V | V | V | V |
| I | I | I | I | I | I | I | I | I | I | I | I |
| D (sample size) | V | D | D (sample size) | V | D | D (sample size) | V | D | D (sample size) | V | D |
| I | I | I | I | I | I | I | I | I | I | I | I |

| Roren et al. | | | Chen and Treleavan (JPE Conventional) | | | Chen and Treleavan (JPE Torsion) | | | Chen and Treleavan (JPE Enbloc) | | |
| --- | --- | --- | --- | --- | --- | --- | --- | --- | --- | --- | --- |
| **rater 1** | **rater 2** | **Consensus** | **rater 1** | **rater 2** | **Consensus** | **rater 1** | **rater 2** | **Consensus** | **rater 1** | **rater 2** | **Consensus** |
| V | V | V | V | V | V | V | V | V | V | V | V |
| V | V | V | I | I | I | I | I | I | I | I | I |
| I (No. of trials, speed of testing) | I (No. of trials, speed of testing) | I | A (sample size) | A (sample size) | A | A (sample size) | A (sample size) | A | A (sample size) | A (sample size) | A |
| I | I | I | I | I | I | I | I | I | I | I | I |
