# Supplementary material for: Measurement properties of cervical joint position error in people with and without neck pain: a systematic review and narrative synthesis
Source: BMC Musculoskelet Disord. 2024 Jan 10;25:44. doi: 10.1186/s12891-023-07111-4 (PMC10777525; doi:10.1186/s12891-023-07111-4)
Supplement: Supplementary file 2 — Additional file 2. [file 12891_2023_7111_MOESM2_ESM.docx]

**Criterion validity – Risk of bias**

| Score: V= very good; A = adequate; D = doubtful; I = inadequate; N= not applicable |
| --- |
|  |
| 1- Were patients stable in the time between the repeated measurements on the construct to be measured? |
| 2- Was the time interval between the repeated measurements appropriate? |
| 3- Were the measurement conditions similar for the repeated measurements – except for the condition being evaluated as a source of variation? |
| 4- Did the professional(s) administer the measurement without knowledge of scores or values of other repeated measurement(s) in the same patients? |
| 5- Did the professional(s) assign scores or determine values without knowledge of the scores or values of other repeated measurement(s) in the same patients? |
| 6- Were there any other important flaws in the design or statistical methods of the study? |
| 7- For continuous scores: was an intraclass correlation coefficient (ICC) calculated? |
| 8- Kappa calculated; the weighting scheme was described, and matches the study design and the data |
| 9- For dichotomous/nominal scores: was Kappa calculated for each category against the other categories combined? |
| Total (lowest score) |

| Chen and Treleavan (JPE conventional) | | | Chen and Treleavan (JPE torsion) | | | Dugailly et al. | | | Roren et al. | | |
| --- | --- | --- | --- | --- | --- | --- | --- | --- | --- | --- | --- |
| Rater 1 | Rater 2 | Consensus | Rater 1 | Rater 2 | Consensus | Rater 1 | Rater 2 | Consensus | Rater 1 | Rater 2 | Consensus |
| A | A | A | A | A | A | A | A | A | A | A | A |
| NA | NA | NA | NA | NA | NA | NA | NA | NA | NA | NA | NA |
| A | A | A | A | A | A | A | A | A | A | A | A |
| A | A | A | A | A | A | A | A | A | A | A | A |
| A | A | A | A | A | A | A | A | A | A | A | A |
| A (sample size) | A | A (sample size) | A (sample size) | A | A (sample size) | D (sample size) | D | D (sample size) | I (No. of trials, speed of testing) | I | I |
| V (correlation calculated | V | V (correlation calculated | V (correlation calculated | V | V (correlation calculated | V (correlation calculated | V | V (correlation calculated | V (correlation calculated | V | V (correlation calculated |
| NA | NA | NA | NA | NA | NA | NA | NA | NA | NA | NA | NA |
| NA | NA | NA | NA | NA | NA | NA | NA | NA | NA | NA | NA |
| A | A | A | A | A | A | D | D | D | I | I | I |

| Wibault et al. | | | Nikkhoo et al. | | |
| --- | --- | --- | --- | --- | --- |
| Rater 1 | Rater 2 | Consensus | Rater 1 | Rater 2 | Consensus |
| A | A | A | A | A | A |
| NA | NA | NA | NA | NA | NA |
| A | A | A | A | A | A |
| A | A | A | A | A | A |
| A | A | A | A | A | A |
| D (sample size) | D | D (sample size) | A (sample size) | A | A (sample size) |
| V (correlation calculated | V | V (correlation calculated | V (correlation calculated | V | V (correlation calculated |
| NA | NA | NA | NA | NA | NA |
| NA | NA | NA | NA | NA | NA |
| D | D | D | A | A | A |
