# Supplementary material for: Measurement properties of cervical joint position error in people with and without neck pain: a systematic review and narrative synthesis
Source: BMC Musculoskelet Disord. 2024 Jan 10;25:44. doi: 10.1186/s12891-023-07111-4 (PMC10777525; doi:10.1186/s12891-023-07111-4)
Supplement: Supplementary file 3 — Additional file 3. [file 12891_2023_7111_MOESM3_ESM.docx]

**Reliability – Risk of bias**

| Score: V= very good; A = adequate; D = doubtful; I = inadequate; N= not applicable |
| --- |
|  |
| 1- Were patients stable in the time between the repeated measurements on the construct to be measured? |
| 2- Was the time interval between the repeated measurements appropriate? |
| 3- Were the measurement conditions similar for the repeated measurements – except for the condition being evaluated as a source of variation? |
| 4- Did the professional(s) administer the measurement without knowledge of scores or values of other repeated measurement(s) in the same patients? |
| 5- Did the professional(s) assign scores or determine values without knowledge of the scores or values of other repeated measurement(s) in the same patients? |
| 6- Were there any other important flaws in the design or statistical methods of the study? |
| 7- For continuous scores: was an intraclass correlation coefficient (ICC) calculated? |
| 8- Kappa calculated; the weighting scheme was described, and matches the study design and the data |
| 9- For dichotomous/nominal scores: was Kappa calculated for each category against the other categories combined? |
| Total (lowest score) |

| **Alahmari et al. (Intra NHP)** | | | **Alahmari et al. (inter NHP)** | | | **Alahmari et al. (Intra THP)** | | | **Alahmari et al. (inter THP)** | | |
| --- | --- | --- | --- | --- | --- | --- | --- | --- | --- | --- | --- |
| Rater 1 | Rater 2 | Consensus | Rater 1 | Rater 2 | Consensus | Rater 1 | Rater 2 | Consensus | Rater 1 | Rater 2 | Consensus |
| A | A | A | A | A | A | A | A | A | A | A | A |
| D (3 days) | D | D (3 days) | D (3 days) | D | D (3 days) | D (3 days) | D | D (3 days) | D (3 days) | D | D (3 days) |
| A | A | A | A | A | A | A | A | A | A | A | A |
| A | A | A | A | A | A | A | A | A | A | A | A |
| A | A | A | A | A | A | A | A | A | A | A | A |
| I (No. of trials | I | I (No. of trials | I (No. of trials | I | I (No. of trials | I (No. of trials | I | I (No. of trials | I (No. of trials | I | I (No. of trials |
| V | V | V | V | V | V | V | V | V | V | V | V |
| NA | NA | NA | NA | NA | NA | NA | NA | NA | NA | NA | NA |
| NA | NA | NA | NA | NA | NA | NA | NA | NA | NA | NA | NA |
| I | I | I | I | I | I | I | I | I | I | I | I |

| **Artz et al. (JPE) within day sitting** | | | **Artz et al. (JPE) between day sitting** | | | **Artz et al. (JPE) within day standing** | | | **Artz et al. (JPE) between day standing** | | |
| --- | --- | --- | --- | --- | --- | --- | --- | --- | --- | --- | --- |
| Rater 1 | Rater 2 | Consensus | Rater 1 | Rater 2 | Consensus | Rater 1 | Rater 2 | Consensus | Rater 1 | Rater 2 | Consensus |
| A | A | A | A | A | A | A | A | A | A | A | A |
| D | D | D | D | D | D | D | D | D | D | D | D |
| A | A | A | A | A | A | A | A | A | A | A | A |
| A | A | A | A | A | A | A | A | A | A | A | A |
| A | A | A | A | A | A | A | A | A | A | A | A |
| I (No. of trials, did not report ICC model ) | I | I (No. of trials, did not report ICC model ) | I (No. of trials, did not report ICC model ) | I | I (No. of trials, did not report ICC model ) | I (No. of trials, did not report ICC model ) | I | I (No. of trials, did not report ICC model ) | I (No. of trials, did not report ICC model ) | I | I (No. of trials, did not report ICC model ) |
| A | V | A | A | A | A | A | A | A | A | A | A |
| NA | NA | NA | NA | NA | NA | NA | NA | NA | NA | NA | NA |
| NA | NA | NA | NA | NA | NA | NA | NA | NA | NA | NA | NA |
| I | I | I | I | I | I | I | I | I | I | I | I |

| **Burke et al. (intra CROM)** | | | **Burke et al. (Intra AL)** | | | **Burke et al. (Inter CROM)** | | | **Burke et al. (Inter AL)** | | |
| --- | --- | --- | --- | --- | --- | --- | --- | --- | --- | --- | --- |
| Rater 1 | Rater 2 | Consensus | Rater 1 | Rater 2 | Consensus | Rater 1 | Rater 2 | Consensus | Rater 1 | Rater 2 | Consensus |
| A | A | A | A | A | A | A | A | A | A | A | A |
| D | D | D | D | D | D | D | D | D | D | D | D |
| A | A | A | A | A | A | A | A | A | A | A | A |
| A | A | A | A | A | A | A | A | A | A | A | A |
| A | A | A | A | A | A | A | A | A | A | A | A |
| I (age not provided and No of trials) | I | I (age not provided and No of trials) | I (age not provided and No of trials) | I | I (age not provided and No of trials) | I (age not provided and No of trials) | I | I (age not provided and No of trials) | I (age not provided and No of trials) | I | I (age not provided and No of trials) |
| V | V | V | V | V | V | V | V | V | V | V | V |
| NA | NA | NA | NA | NA | NA | NA | NA | NA | NA | NA | NA |
| NA | NA | NA | NA | NA | NA | NA | NA | NA | NA | NA | NA |
| I | I | I | I | I | I | I | I | I | I | I | I |

| **Goncalves and Silva (within day HRNT)** | | | **Goncalves and Silva (within day TT)** | | | **Goncalves and Silva (within day HR30T)** | | | **Goncalves and Silva (within day F8T)** | | |
| --- | --- | --- | --- | --- | --- | --- | --- | --- | --- | --- | --- |
| Rater 1 | Rater 2 | Consensus | Rater 1 | Rater 2 | Consensus | Rater 1 | Rater 2 | Consensus | Rater 1 | Rater 2 | Consensus |
| A | A | A | A | A | A | A | A | A | A | A | A |
| D | D | D | D | D | D | D | D | D | D | D | D |
| A | A | A | A | A | A | A | A | A | A | A | A |
| A | A | A | A | A | A | A | A | A | A | A | A |
| A | A | A | A | A | A | A | A | A | A | A | A |
| V | V | V | V | V | V | V | V | V | V | V | V |
| V | V | V | V | V | V | V | V | V | V | V | V |
| NA | NA | NA | NA | NA | NA | NA | NA | NA | NA | NA | NA |
| NA | NA | NA | NA | NA | NA | NA | NA | NA | NA | NA | NA |
| D | D | D | D | D | D | D | D | D | D | D | D |

| **Goncalves and Silva (between day HRNT)** | | | **Goncalves and Silva (between day TT)** | | | **Goncalves and Silva (between day HR30T)** | | | **Goncalves and Silva ( between day F8T)** | | |
| --- | --- | --- | --- | --- | --- | --- | --- | --- | --- | --- | --- |
| Rater 1 | Rater 2 | Consensus | Rater 1 | Rater 2 | Consensus | Rater 1 | Rater 2 | Consensus | Rater 1 | Rater 2 | Consensus |
| A | A | A | A | A | A | A | A | A | A | A | A |
| D | D | D | D | D | D | D | D | D | D | D | D |
| A | A | A | A | A | A | A | A | A | A | A | A |
| A | A | A | A | A | A | A | A | A | A | A | A |
| A | A | A | A | A | A | A | A | A | A | A | A |
| V | V | V | V | V | V | V | V | V | V | V | V |
| V | V | V | V | V | V | V | V | V | V | V | V |
| NA | NA | NA | NA | NA | NA | NA | NA | NA | NA | NA | NA |
| NA | NA | NA | NA | NA | NA | NA | NA | NA | NA | NA | NA |
| D | D | D | D | D | D | D | D | D | D | D | D |

| **Kristjansson et al. 2001 (NHP)** | | | **Kristjansson et al. 2001 (THP)** | | | **Kristjansson et al. 2001 (preset trunk rotation)** | | | **Kristjansson et al. 2001 (Fo8 relocation test)** | | |
| --- | --- | --- | --- | --- | --- | --- | --- | --- | --- | --- | --- |
| Rater 1 | Rater 2 | Consensus | Rater 1 | Rater 2 | Consensus | Rater 1 | Rater 2 | Consensus | Rater 1 | Rater 2 | Consensus |
| A | A | A | A | A | A | A | A | A | A | A | A |
| D | D | D | D | D | D | D | D | D | D | D | D |
| A | A | A | A | A | A | A | A | A | A | A | A |
| A | A | A | A | A | A | A | A | A | A | A | A |
| A | A | A | A | A | A | A | A | A | A | A | A |
| I (sample size and No. of trials) | I | I (sample size and No. of trials) | I (sample size and No. of trials) | I | I (sample size and No. of trials) | I (sample size and No. of trials) | I | I (sample size and No. of trials) | I (sample size and No. of trials) | I | I (sample size and No. of trials) |
| V | V | V | V | V | V | V | V | V | V | V | V |
| NA | NA | NA | NA | NA | NA | NA | NA | NA | NA | NA | NA |
| NA | NA | NA | NA | NA | NA | NA | NA | NA | NA | NA | NA |
| I | I | I | I | I | I | I | I | I | I | I | I |

| **Lee et al. (NHP)** | | | **Lee et al. (THP)** | | | **Pinsault et al.** | | | **Strimpakos et al. (intrarater sitting)** | | |
| --- | --- | --- | --- | --- | --- | --- | --- | --- | --- | --- | --- |
| Rater 1 | Rater 2 | Consensus | Rater 1 | Rater 2 | Consensus | Rater 1 | Rater 2 | Consensus | Rater 1 | Rater 2 | Consensus |
| A | A | A | A | A | A | A | A | A | A | A | A |
| D | D | D | D | D | D | D | D | D | D | D | D |
| A | A | A | A | A | A | A | A | A | A | A | A |
| A | A | A | A | A | A | A | A | A | A | A | A |
| A | A | A | A | A | A | A | A | A | A | A | A |
| I (No. of trials, no randomisation, sample size) | I | I (No. of trials, no randomisation, sample size) | I (No. of trials, no randomisation, sample size) | I | I (No. of trials, no randomisation, sample size) | V | V | V | I (No. of trials, no randomisation) | I | I (No. of trials, no randomisation) |
| V | V | V | V | V | V | V | V | V | V | V | V |
| NA | NA | NA | NA | NA | NA | NA | NA | NA | NA | NA | NA |
| NA | NA | NA | NA | NA | NA | NA | NA | NA | NA | NA | NA |
| I | I | I | I | I | I | D | D | D | I | I | I |

| **Strimpakos et al. (interrater)** | | | **Wibault et al.** | | | **Roren (Intrarater within day Revel visual technique)** | | | **Roren (Intrarater within day US technique)** | | |
| --- | --- | --- | --- | --- | --- | --- | --- | --- | --- | --- | --- |
| Rater 1 | Rater 2 | Consensus | Rater 1 | Rater 2 | Consensus | Rater 1 | Rater 2 | Consensus | Rater 1 | Rater 2 | Consensus |
| A | A | A | A | A | A | A | A | A | A | A | A |
| D | D | D | D | D | D | D | D | D | D | D | D |
| A | A | A | A | A | A | A | A | A | A | A | A |
| A | A | A | A | A | A | A | A | A | A | A | A |
| A | A | A | A | A | A | A | A | A | A | A | A |
| I (No. of trials, no randomisation) | I | I (No. of trials, no randomisation) | I (No. of trials) | I | I (No. of trials) | I (No. of trials, speed of testing, did not report ICC model) | I | I (No. of trials, speed of testing, did not report ICC model) | I (No. of trials, speed of testing, did not report ICC model) | I | I (No. of trials, speed of testing, did not report ICC model) |
| V | V | V | A | A | A | V | V | V | V | V | V |
| NA | NA | NA | NA | NA | NA | NA | NA | NA | NA | NA | NA |
| NA | NA | NA | NA | NA | NA | NA | NA | NA | NA | NA | NA |
| I | I | I | I | I | I | I | I | I | I | I | I |

| **Nikkhoo (within day intra US MOCAP)** | | | **Nikkhoo (between day intra US MOCAP)** | | | **Nikkhoo (within day intra IMU)** | | | **Nikkhoo (between day intra IMU)** | | |
| --- | --- | --- | --- | --- | --- | --- | --- | --- | --- | --- | --- |
| Rater 1 | Rater 2 | Consensus | Rater 1 | Rater 2 | Consensus | Rater 1 | Rater 2 | Consensus | Rater 1 | Rater 2 | Consensus |
| A | A | A | A | A | A | A | A | A | A | A | A |
| D | D | D | D | D | D | D | D | D | D | D | D |
| A | A | A | A | A | A | A | A | A | A | A | A |
| A | A | A | A | A | A | A | A | A | A | A | A |
| A | A | A | A | A | A | A | A | A | A | A | A |
| A (sample size) | A | A (sample size) | A (sample size) | A | A (sample size) | A (sample size) | A | A (sample size) | A (sample size) | A | A (sample size) |
| V | V | V | V | V | V | V | V | V | V | V | V |
| NA | NA | NA | NA | NA | NA | NA | NA | NA | NA | NA | NA |
| NA | NA | NA | NA | NA | NA | NA | NA | NA | NA | NA | NA |
| D | D | D | D | D | D | D | D | D | D | D | D |

| **Strimpakos et al. (intrarater standing)** | | | **Cid et al.** | | | **Kramer et al. (intrasession)** | | | **Kramer et al. (intersession)** | | |
| --- | --- | --- | --- | --- | --- | --- | --- | --- | --- | --- | --- |
| Rater 1 | Rater 2 | Consensus | Rater 1 | Rater 2 | Consensus | Rater 1 | Rater 2 | Consensus | Rater 1 | Rater 2 | Consensus |
| A | A | A | A | A | A | A | A | A | A | A | A |
| D | D | D | D | D | D | D | D | D | D | D | D |
| A | A | A | A | A | A | A | A | A | A | A | A |
| A | A | A | A | A | A | A | A | A | A | A | A |
| A | A | A | A | A | A | A | A | A | A | A | A |
| I (No. of trials, no randomisation) | I | I (No. of trials, no randomisation) | V | V | V | I (no randomisation, NO. of trials) | I | I (no randomisation, NO. of trials) | I (no randomisation, NO. of trials) | I | I (no randomisation, NO. of trials) |
| V | V | V | V | V | V | V | V | V | V | V | V |
| NA | NA | NA | NA | NA | NA | NA | NA | NA | NA | NA | NA |
| NA | NA | NA | NA | NA | NA | NA | NA | NA | NA | NA | NA |
| I | I | I | D | D | D | D | D | D | D | D | D |

| **Dugailly et al. (90cm low speed)** | | | **Dugailly et al. (90cm high speed)** | | | **Dugailly et al. (180cm low speed)** | | | **Dugailly et al. (180cm high speed)** | | |
| --- | --- | --- | --- | --- | --- | --- | --- | --- | --- | --- | --- |
| Rater 1 | Rater 2 | Consensus | Rater 1 | Rater 2 | Consensus | Rater 1 | Rater 2 | Consensus | Rater 1 | Rater 2 | Consensus |
| A | A | A | A | A | A | A | A | A | A | A | A |
| D | D | D | D | D | D | D | D | D | D | D | D |
| A | A | A | A | A | A | A | A | A | A | A | A |
| A | A | A | A | A | A | A | A | A | A | A | A |
| A | A | A | A | A | A | A | A | A | A | A | A |
| I (sample size, did not report ICC model) | I | I (sample size, did not report ICC model) | I (sample size, did not report ICC model) | I | I (sample size, did not report ICC model) | I (sample size, did not report ICC model) | I | I (sample size, did not report ICC model) | I (sample size, did not report ICC model) | I | I (sample size, did not report ICC model) |
| V | V | V | V | V | V | V | V | V | V | V | V |
| NA | NA | NA | NA | NA | NA | NA | NA | NA | NA | NA | NA |
| NA | NA | NA | NA | NA | NA | NA | NA | NA | NA | NA | NA |
| I | I | I | I | I | I | I | I | I | I | I | I |

**Measurement error**

| Score: V= very good; A = adequate; D = doubtful; I = inadequate; N= not applicable |
| --- |
|  |
| 1- Were patients stable in the time between the repeated measurements on the construct to be measured? |
| 2- Was the time interval between the repeated measurements appropriate? |
| 3- Were the measurement conditions similar for the repeated measurements – except for the condition being evaluated as a source of variation? |
| 4- Did the professional(s) administer the measurement without knowledge of scores or values of other repeated measurement(s) in the same patients? |
| 5- Did the professional(s) assign scores or determine values without knowledge of the scores or values of other repeated measurement(s) in the same patients? |
| 6- Were there any other important flaws in the design or statistical methods of the study? |
| 7- For continuous scores: was the Standard Error of Measurement (SEM), Smallest Detectable Change (SDC), Limits of Agreement (LoA) or Coefficient of Variation (CV) calculated? |
| 8- For dichotomous/nominal/ordinal scores: Was the percentage specific (e.g. positive and negative) agreement calculated? |
| Total (lowest score) |

| **Alahmari et al. (Intra NHP)** | | | **Alahmari et al. (inter NHP)** | | | **Alahmari et al. (Intra THP)** | | | **Alahmari et al. (inter THP)** | | |
| --- | --- | --- | --- | --- | --- | --- | --- | --- | --- | --- | --- |
| Rater 1 | Rater 2 | Consensus | Rater 1 | Rater 2 | Consensus | Rater 1 | Rater 2 | Consensus | Rater 1 | Rater 2 | Consensus |
| A | A | A | A | A | A | A | A | A | A | A | A |
| D | D | D | D | D | D | D | D | D | D | D | D |
| A | A | A | A | A | A | A | A | A | A | A | A |
| A | A | A | A | A | A | A | A | A | A | A | A |
| A | A | A | A | A | A | A | A | A | A | A | A |
| I (No. of trials | I | I (No. of trials | I (No. of trials | I | I (No. of trials | I (No. of trials | I | I (No. of trials | I (No. of trials | I | I (No. of trials |
| V | V | V | V | V | V | V | V | V | V | V | V |
| NA | NA | NA | NA | NA | NA | NA | NA | NA | NA | NA | NA |
| I | I | I | I | I | I | I | I | I | I | I | I |

| **Artz et al. (JPE) within day sitting** | | | **Artz et al. (JPE) between day sitting** | | | **Artz et al. (JPE) within day standing** | | | **Artz et al. (JPE) between day standing** | | |
| --- | --- | --- | --- | --- | --- | --- | --- | --- | --- | --- | --- |
| Rater 1 | Rater 2 | Consensus | Rater 1 | Rater 2 | Consensus | Rater 1 | Rater 2 | Consensus | Rater 1 | Rater 2 | Consensus |
| A | A | A | A | A | A | A | A | A | A | A | A |
| D | D | D | D | D | D | D | D | D | D | D | D |
| A | A | A | A | A | A | A | A | A | A | A | A |
| A | A | A | A | A | A | A | A | A | A | A | A |
| A | A | A | A | A | A | A | A | A | A | A | A |
| I (No. of trials ) | I | I (No. of trials ) | I (No. of trials ) | I | I (No. of trials ) | I (No. of trials ) | I | I (No. of trials ) | I (No. of trials ) | I | I (No. of trials ) |
| V | V | V | V | V | V | V | V | V | V | V | V |
| NA | NA | NA | NA | NA | NA | NA | NA | NA | NA | NA | NA |
| I | I | I | I | I | I | I | I | I | I | I | I |

| **Burke et al. (intra CROM)** | | | **Burke et al. (Intra AL)** | | | **Burke et al. (Inter CROM)** | | | **Burke et al. (Inter AL)** | | |
| --- | --- | --- | --- | --- | --- | --- | --- | --- | --- | --- | --- |
| Rater 1 | Rater 2 | Consensus | Rater 1 | Rater 2 | Consensus | Rater 1 | Rater 2 | Consensus | Rater 1 | Rater 2 | Consensus |
| A | A | A | A | A | A | A | A | A | A | A | A |
| D | D | D | D | D | D | D | D | D | D | D | D |
| A | A | A | A | A | A | A | A | A | A | A | A |
| A | A | A | A | A | A | A | A | A | A | A | A |
| A | A | A | A | A | A | A | A | A | A | A | A |
| I (age not provided and No of trials) | I | I (age not provided and No of trials) | I (age not provided and No of trials) | I | I (age not provided and No of trials) | I (age not provided and No of trials) | I | I (age not provided and No of trials) | I (age not provided and No of trials) | I | I (age not provided and No of trials) |
| V | V | V | V | V | V | V | V | V | V | V | V |
| NA | NA | NA | NA | NA | NA | NA | NA | NA | NA | NA | NA |
| I | I | I | I | I | I | I | I | I | I | I | I |

| **Goncalves and Silva (within day HRNT)** | | | **Goncalves and Silva (within day TT)** | | | **Goncalves and Silva (within day HR30T)** | | | **Goncalves and Silva (within day F8T)** | | |
| --- | --- | --- | --- | --- | --- | --- | --- | --- | --- | --- | --- |
| Rater 1 | Rater 2 | Consensus | Rater 1 | Rater 2 | Consensus | Rater 1 | Rater 2 | Consensus | Rater 1 | Rater 2 | Consensus |
| A | A | A | A | A | A | A | A | A | A | A | A |
| D | D | D | D | D | D | D | D | D | D | D | D |
| A | A | A | A | A | A | A | A | A | A | A | A |
| A | A | A | A | A | A | A | A | A | A | A | A |
| A | A | A | A | A | A | A | A | A | A | A | A |
| V | V | V | V | V | V | V | V | V | V | V | V |
| V | V | V | V | V | V | V | V | V | V | V | V |
| NA | NA | NA | NA | NA | NA | NA | NA | NA | NA | NA | NA |
| D | D | D | D | D | D | D | D | D | D | D | D |

| **Goncalves and Silva (between day HRNT)** | | | **Goncalves and Silva (between day TT)** | | | **Goncalves and Silva (between day HR30T)** | | | **Goncalves and Silva ( between day F8T)** | | |
| --- | --- | --- | --- | --- | --- | --- | --- | --- | --- | --- | --- |
| Rater 1 | Rater 2 | Consensus | Rater 1 | Rater 2 | Consensus | Rater 1 | Rater 2 | Consensus | Rater 1 | Rater 2 | Consensus |
| A | A | A | A | A | A | A | A | A | A | A | A |
| D | D | D | D | D | D | D | D | D | D | D | D |
| A | A | A | A | A | A | A | A | A | A | A | A |
| A | A | A | A | A | A | A | A | A | A | A | A |
| A | A | A | A | A | A | A | A | A | A | A | A |
| V | V | V | V | V | V | V | V | V | V | V | V |
| V | V | V | V | V | V | V | V | V | V | V | V |
| NA | NA | NA | NA | NA | NA | NA | NA | NA | NA | NA | NA |
| D | D | D | D | D | D | D | D | D | D | D | D |

| **Kristjansson et al. 2001 (NHP)** | | | **Kristjansson et al. 2001 (THP)** | | | **Kristjansson et al. 2001 (preset trunk rotation)** | | | **Kristjansson et al. 2001 (Fo8 relocation test)** | | |
| --- | --- | --- | --- | --- | --- | --- | --- | --- | --- | --- | --- |
| Rater 1 | Rater 2 | Consensus | Rater 1 | Rater 2 | Consensus | Rater 1 | Rater 2 | Consensus | Rater 1 | Rater 2 | Consensus |
| A | A | A | A | A | A | A | A | A | A | A | A |
| D | D | D | D | D | D | D | D | D | D | D | D |
| A | A | A | A | A | A | A | A | A | A | A | A |
| A | A | A | A | A | A | A | A | A | A | A | A |
| A | A | A | A | A | A | A | A | A | A | A | A |
| I (sample size and No. of trials) | I | I (sample size and No. of trials) | I (sample size and No. of trials) | I | I (sample size and No. of trials) | I (sample size and No. of trials) | I | I (sample size and No. of trials) | I (sample size and No. of trials) | I | I (sample size and No. of trials) |
| V | V | V | V | V | V | V | V | V | V | V | V |
| NA | NA | NA | NA | NA | NA | NA | NA | NA | NA | NA | NA |
| I | I | I | I | I | I | I | I | I | I | I | I |

| **Lee et al. (NHP)** | | | **Lee et al. (THP)** | | | **Pinsault et al.** | | | **Strimpakos et al. (intrarater sitting)** | | |
| --- | --- | --- | --- | --- | --- | --- | --- | --- | --- | --- | --- |
| Rater 1 | Rater 2 | Consensus | Rater 1 | Rater 2 | Consensus | Rater 1 | Rater 2 | Consensus | Rater 1 | Rater 2 | Consensus |
| A | A | A | A | A | A | A | A | A | A | A | A |
| D | D | D | D | D | D | D | D | D | D | D | D |
| A | A | A | A | A | A | A | A | A | A | A | A |
| A | A | A | A | A | A | A | A | A | A | A | A |
| A | A | A | A | A | A | A | A | A | A | A | A |
| I (No. of trials, no randomisation) | I | I (No. of trials, no randomisation) | I (No. of trials, no randomisation) | I | I (No. of trials, no randomisation) | V | V | V | I (No. of trials, no randomisation) | I | I (No. of trials, no randomisation) |
| V | V | V | V | V | V | V | V | V | V | V | V |
| NA | NA | NA | NA | NA | NA | NA | NA | NA | NA | NA | NA |
| I | I | I | I | I | I | D | D | D | I | I | I |

| **Strimpakos et al. (interrater)** | | | **Wibault et al.** | | | **Roren (Intrarater within day Revel visual technique)** | | | **Roren (Intrarater within day US technique)** | | |
| --- | --- | --- | --- | --- | --- | --- | --- | --- | --- | --- | --- |
| Rater 1 | Rater 2 | Consensus | Rater 1 | Rater 2 | Consensus | Rater 1 | Rater 2 | Consensus | Rater 1 | Rater 2 | Consensus |
| A | A | A | A | A | A | A | A | A | A | A | A |
| D | D | D | D | D | D | D | D | D | D | D | D |
| A | A | A | A | A | A | A | A | A | A | A | A |
| A | A | A | A | A | A | A | A | A | A | A | A |
| A | A | A | A | A | A | A | A | A | A | A | A |
| I (No. of trials, no randomisation) | I | I (No. of trials, no randomisation) | I (No. of trials) | I | I (No. of trials) | I (No. of trials, speed of testing) | I (No. of trials, speed of testing) | I (No. of trials, speed of testing) | I (No. of trials, speed of testing) | I (No. of trials, speed of testing) | I (No. of trials, speed of testing) |
| V | V | V | V | V | V | V | V | V | V | V | V |
| NA | NA | NA | NA | NA | NA | NA | NA | NA | NA | NA | NA |
| I | I | I | I | I | I | I | I | I | I | I | I |

| **Nikkhoo (within day intra US MOCAP)** | | | **Nikkhoo (between day intra US MOCAP)** | | | **Nikkhoo (within day intra IMU)** | | | **Nikkhoo (between day intra IMU)** | | |
| --- | --- | --- | --- | --- | --- | --- | --- | --- | --- | --- | --- |
| Rater 1 | Rater 2 | Consensus | Rater 1 | Rater 2 | Consensus | Rater 1 | Rater 2 | Consensus | Rater 1 | Rater 2 | Consensus |
| A | A | A | A | A | A | A | A | A | A | A | A |
| D | D | D | D | D | D | D | D | D | D | D | D |
| A | A | A | A | A | A | A | A | A | A | A | A |
| A | A | A | A | A | A | A | A | A | A | A | A |
| A | A | A | A | A | A | A | A | A | A | A | A |
| A | A | A | A | A | A | A | A | A | A | A | A |
| V | V | V | V | V | V | V | V | V | V | V | V |
| NA | NA | NA | NA | NA | NA | NA | NA | NA | NA | NA | NA |
| D | D | D | D | D | D | D | D | D | D | D | D |

| **Strimpakos et al. (intrarater standing)** | | | **Dugailly et al. (90cm low speed)** | | | **Dugailly et al. (90cm high speed)** | | | **Dugailly et al. (180cm low speed)** | | | **Dugailly et al. (180cm high speed)** | | |
| --- | --- | --- | --- | --- | --- | --- | --- | --- | --- | --- | --- | --- | --- | --- |
| Rater 1 | Rater 2 | Consensus | Rater 1 | Rater 2 | Consensus | Rater 1 | Rater 2 | Consensus | Rater 1 | Rater 2 | Consensus | Rater 1 | Rater 2 | Consensus |
| A | A | A | A | A | A | A | A | A | A | A | A | A | A | A |
| D | D | D | D | D | D | D | D | D | D | D | D | D | D | D |
| A | A | A | A | A | A | A | A | A | A | A | A | A | A | A |
| A | A | A | A | A | A | A | A | A | A | A | A | A | A | A |
| A | A | A | A | A | A | A | A | A | A | A | A | A | A | A |
| I (No. of trials, no randomisation) | I | I (No. of trials, no randomisation) | I (sample size) | I | I (sample size) | I (sample size) | I | I (sample size) | I (sample size) | I | I (sample size) | I (sample size) | I | I (sample size) |
| V | V | V | V | V | V | V | V | V | V | V | V | V | V | V |
| NA | NA | NA | NA | NA | NA | NA | NA | NA | NA | NA | NA | NA | NA | NA |
| I | I | I | I | I | I | I | I | I | I | I | I | I | I | I |
