# Supplementary material for: Measurement properties of cervical joint position error in people with and without neck pain: a systematic review and narrative synthesis
Source: BMC Musculoskelet Disord. 2024 Jan 10;25:44. doi: 10.1186/s12891-023-07111-4 (PMC10777525; doi:10.1186/s12891-023-07111-4)
Supplement: Supplementary file 4 — Additional file 4. [file 12891_2023_7111_MOESM4_ESM.docx]

**The overall level of evidence for each outcome measure and its respective measurement property using a modified Grading of Recommendations Assessment, Development, and Evaluation (GRADE) approach.**

**NP (AE) (NHP) (Table 5 in manuscript)**

| Reference | Intrarater reliability | | | Interrater reliability | | | Measurement error | | | Convergent validity | | | Discriminative validity | | | Criterion validity | | |
| --- | --- | --- | --- | --- | --- | --- | --- | --- | --- | --- | --- | --- | --- | --- | --- | --- | --- | --- |
|  | n | Meth. Qual. | Rating | n | Meth. Qual. | Rating | n | Meth. Qual. | Rating | n | Meth. Qual. | Rating | n | Meth. Qual. | Rating | n | Meth. Qual. | Rating |
| Alahmari et al. (intra NHP) | 69 | I | (+) (ICC: 0.74-0.78) |  |  |  | 69 | I | (?) | #SPILL! |  |  |  |  |  |  |  |  |
| Alahmari et al. (inter NHP) |  |  |  | 69 | I | (+) (ICC: 0.74-0.79) | 69 | I | (?) |  |  |  |  |  |  |  |  |  |
| Burke et al. (intra CROM) | 50 | I | (-) (ICC: 0.25-0.38) |  |  |  | 50 | I | (?) |  |  |  |  |  |  |  |  |  |
| Burke et al. (intra AL) | 50 | I | (-) (ICC: 0.48-0.55) |  |  |  | 50 | I | (?) |  |  |  |  |  |  |  |  |  |
| Burke et al. (inter CROM) |  |  |  | 50 | I | (+) (ICC: 0.71-0.77) | 50 | I | (?) |  |  |  |  |  |  |  |  |  |
| Burke et al. (inter AL) |  |  |  | 50 | I | (+) (ICC: 0.58-0.75) | 50 | I | (?) |  |  |  |  |  |  |  |  |  |
| Chen and Treleavan (JPE Conventional) |  |  |  |  |  |  |  |  |  |  |  |  | 50 | I | (?) | 51 | A | (+) (r=0.87) |
| Chen and Treleavan (JPE Torion) |  |  |  |  |  |  |  |  |  |  |  |  | 50 | I | (?) | 51 | A | (-) (r=0.67) |
| Chen and Treleavan (JPE Enbloc) |  |  |  |  |  |  |  |  |  |  |  |  | 50 | I | (?) |  |  |  |
| Chen and Treleavan (JPE Conventional vs NDI) |  |  |  |  |  |  |  |  |  | 51 | A | (-) |  |  |  |  |  |  |
| Chen and Treleavan (JPE Torion vs NDI) |  |  |  |  |  |  |  |  |  | 51 | A | (-) |  |  |  |  |  |  |
| Chen and Treleavan (JPE Enbloc vs NDI) |  |  |  |  |  |  |  |  |  | 51 | A | (-) |  |  |  |  |  |  |
| Chen and Treleavan (JPE Conventional vs VAS) |  |  |  |  |  |  |  |  |  | 51 | A | (+) (r=0.51) |  |  |  |  |  |  |
| Chen and Treleavan (JPE Torion vs VAS) |  |  |  |  |  |  |  |  |  | 51 | A | (-) |  |  |  |  |  |  |
| Chen and Treleavan (JPE Enbloc vs VAS) |  |  |  |  |  |  |  |  |  | 51 | A | (-) |  |  |  |  |  |  |
| Goncalves and Silva (intra within day HRNT) | 33 | D | (+) (ICC: 0.9-0.93) |  |  |  | 33 | D | (?) |  |  |  |  |  |  |  |  |  |
| Goncalves and Silva (intra within day TT) | 33 | D | (+) (ICC: 0.88-0.9) |  |  |  | 33 | D | (?) |  |  |  |  |  |  |  |  |  |
| Goncalves and Silva (intra within day F8T) | 33 | D | (+) (ICC: 0.89-0.93) |  |  |  | 33 | D | (?) |  |  |  |  |  |  |  |  |  |
| Goncalves and Silva (intra between day HRNT) | 33 | D | (+) (ICC: 0.61-0.85) |  |  |  | 33 | D | (?) |  |  |  |  |  |  |  |  |  |
| Goncalves and Silva (intra between day TT) | 33 | D | (+) (ICC: 0.58-0.71) |  |  |  | 33 | D | (?) |  |  |  |  |  |  |  |  |  |
| Goncalves and Silva (intra between day F8T) | 33 | D | (+) (ICC: 0.66-0.85) |  |  |  | 33 | D | (?) |  |  |  |  |  |  |  |  |  |
| Goncalves and Silva (HRNT vs disability) |  |  |  |  |  |  |  |  |  | 66 | A | (-) (r<0.3) |  |  |  |  |  |  |
| Goncalves and Silva (HRNT vs pain catastrophising) |  |  |  |  |  |  |  |  |  | 66 | A | (-) (r<0.3) |  |  |  |  |  |  |
| Goncalves and Silva (HRNT vs fear of movement |  |  |  |  |  |  |  |  |  | 66 | A | (-) (r<0.3) |  |  |  |  |  |  |
| Goncalves and Silva (TT vs disability) |  |  |  |  |  |  |  |  |  | 66 | A | (-) (r<0.3) |  |  |  |  |  |  |
| Goncalves and Silva (TT vs disability) |  |  |  |  |  |  |  |  |  | 66 | A | (-) (r<0.3) |  |  |  |  |  |  |
| Goncalves and Silva (TT vs disability) |  |  |  |  |  |  |  |  |  | 66 | A | (-) (r<0.3) |  |  |  |  |  |  |
| Goncalves and Silva (HR30T vs disability) |  |  |  |  |  |  |  |  |  | 66 | A | (-) (r<0.3) |  |  |  |  |  |  |
| Goncalves and Silva (HR30T vs disability) |  |  |  |  |  |  |  |  |  | 66 | A | (-) (r<0.3) |  |  |  |  |  |  |
| Goncalves and Silva (HR30T vs disability) |  |  |  |  |  |  |  |  |  | 66 | A | (-) (r<0.3) |  |  |  |  |  |  |
| Goncalves and Silva (F8T vs disability) |  |  |  |  |  |  |  |  |  | 66 | A | (-) (r<0.3) |  |  |  |  |  |  |
| Goncalves and Silva (F8T vs disability) |  |  |  |  |  |  |  |  |  | 66 | A | (-) (r<0.3) |  |  |  |  |  |  |
| Goncalves and Silva (F8T vs disability) |  |  |  |  |  |  |  |  |  | 66 | A | (-) (r<0.3) |  |  |  |  |  |  |
| Goncalves and Silva (HRNT vs TT) |  |  |  |  |  |  |  |  |  | 66 | A | (+) (r=0.35-0.61) |  |  |  |  |  |  |
| Goncalves and Silva (HRNT vs HR30T) |  |  |  |  |  |  |  |  |  | 66 | A | (+) (r=0.35-0.61) |  |  |  |  |  |  |
| Goncalves and Silva (HRNT vs F8T) |  |  |  |  |  |  |  |  |  | 66 | A | (+) (r=0.35-0.61) |  |  |  |  |  |  |
| Goncalves and Silva (TT vs HRNT) |  |  |  |  |  |  |  |  |  | 66 | A | (+) (r=0.35-0.61) |  |  |  |  |  |  |
| Goncalves and Silva (TT vs HR30T) |  |  |  |  |  |  |  |  |  | 66 | A | (+) (r=0.35-0.61) |  |  |  |  |  |  |
| Goncalves and Silva (TT vs F8T) |  |  |  |  |  |  |  |  |  | 66 | A | (+) (r=0.35-0.61) |  |  |  |  |  |  |
| Goncalves and Silva (HR30T vs HRNT) |  |  |  |  |  |  |  |  |  | 66 | A | (+) (r=0.35-0.61) |  |  |  |  |  |  |
| Goncalves and Silva (HR30T vs TT) |  |  |  |  |  |  |  |  |  | 66 | A | (+) (r=0.35-0.61) |  |  |  |  |  |  |
| Goncalves and Silva (HR30T vs F8T) |  |  |  |  |  |  |  |  |  | 66 | A | (+) (r=0.35-0.61) |  |  |  |  |  |  |
| Goncalves and Silva (F8T vs HRNT) |  |  |  |  |  |  |  |  |  | 66 | A | (+) (r=0.35-0.61) |  |  |  |  |  |  |
| Goncalves and Silva (F8T vs TT) |  |  |  |  |  |  |  |  |  | 66 | A | (+) (r=0.35-0.61) |  |  |  |  |  |  |
| Goncalves and Silva (F8T vs HR30T) |  |  |  |  |  |  |  |  |  | 66 | A | (+) (r=0.35-0.61) |  |  |  |  |  |  |
| Goncalves and Silva (HRNT) |  |  |  |  |  |  |  |  |  |  |  |  | 66 | I | (?) |  |  |  |
| Goncalves and Silva (TT) |  |  |  |  |  |  |  |  |  |  |  |  | 66 | I | (?) |  |  |  |
| Goncalves and Silva (HR30T) |  |  |  |  |  |  |  |  |  |  |  |  | 66 | I | (?) |  |  |  |
| Goncalves and Silva (F8T) |  |  |  |  |  |  |  |  |  |  |  |  | 66 | I | (?) |  |  |  |
| Roren et al. (intra Revel visual technique) | 82 | I | (-) (ICC: 0.68) |  |  |  | 82 | I | (?) |  |  |  |  |  |  |  |  |  |
| Roren et al. (intra US technique) | 82 | I | (-) (ICC: 0.62) |  |  |  | 82 | I | (?) |  |  |  |  |  |  |  |  |  |
| Roren (US technique) |  |  |  |  |  |  |  |  |  |  |  |  |  |  |  |  |  |  |
| Roren et al. (validity) |  |  |  |  |  |  |  |  |  |  |  |  | 82 | I | (+) Kappa 0.65 | 82 | I | (+) (r=0.94-0.95) |
| Wibault et al. | 36 | I | (+) (ICC: 0.79-0.85) |  |  |  | 36 | I | (?) |  |  |  |  |  |  |  |  |  |
| Cid et al. | 13 | I | (+) (ICC: 0.77-0.86) |  |  |  |  |  |  |  |  |  |  |  |  |  |  |  |
| Pooled or summary results | 580 | I | (+) (ICC: 0.58-0.93) | 169 | I | (+) (0.58-0.79) | 736 | I | (?) | 1890 | A | (-) (r<0.5) | 496 | I | (?) | 184 | A | (+) (r=0.87-0.95) |
| GRADE (with reason) | Very low (-2 risk of bias, -2 inconsistency) | | | Low (-2 risk of bias) | | | Not graded | | | Low (-2 inconsistency) | | | Very Low (-2 risk of bias,-2 inconsistency) | | | low (-2 inconsistency) | | |

**NP (AE) (THP) (Table 5 in manuscript)**

| Reference | Intrarater reliability | | | Interrater reliability | | | Measurement error | | |
| --- | --- | --- | --- | --- | --- | --- | --- | --- | --- |
|  | n | Meth. Qual. | Rating | n | Meth. Qual. | Rating | n | Meth. Qual. | Rating |
| Alahmari et al. (intra THP) | 69 | I | (+) (ICC: 0.7-0.83) |  |  |  | 69 | I | (?) |
| Alahmari et al. (inter THP) |  |  |  | 69 | I | (+) (ICC: 0.62-0.84) | 69 | I | (?) |
| Goncalves and Silva (intra within day HR30T) | 33 | D | (+) (ICC: 0.73-0.79) |  |  |  | 33 | D | (?) |
| Goncalves and Silva (intra between day HR30T) | 33 | D | (+) (ICC: 0.67-0.7) |  |  |  | 33 | D | (?) |
| Pooled or summary results | 135 | D | (+) (ICC: 0.67-0.83) | 69 | I | (+) (0.58-0.84) | 204 | I | (?) |
| GRADE (with reason) | Low (-2 risk of bias) | | | very low (-3 risk of bias, -1 imprecision) | | | Not graded | | |

**Healthy (AE) (NHP) (Table 5 in manuscript)**

| Reference | Intrarater reliability | | | Interrater reliability | | | Measurement error | | | Criterion validity | | | Intrasession reliability | | | Intersession reliability | | |
| --- | --- | --- | --- | --- | --- | --- | --- | --- | --- | --- | --- | --- | --- | --- | --- | --- | --- | --- |
|  | n | Meth. Qual. | Rating | n | Meth. Qual. | Rating | n | Meth. Qual. | Rating | n | Meth. Qual. | Rating | n | Meth. Qual. | Rating | n | Meth. Qual. | Rating |
| Kristjansson et al. 2001 (NHP) | 19 | I | (-) (ICC: 0.35-0.44) |  |  |  | 19 | I | (?) |  |  |  |  |  |  |  |  |  |
| Kristjansson et al. 2001 (preset trunk rotation) | 19 | I | (+) (ICC: 0.52-0.74) |  |  |  | 19 | I | (?) |  |  |  |  |  |  |  |  |  |
| Kristjansson et al. 2001 (F8 relocation test) | 19 | I | (-) (ICC: 0.67) |  |  |  | 19 | I | (?) |  |  |  |  |  |  |  |  |  |
| Pinsault et al. | 44 | D | (+) (ICC: 0.52-0.81) |  |  |  | 44 | D | (?) |  |  |  |  |  |  |  |  |  |
| Strimpakos et al. (intra sitting) | 35 | I | (-) (ICC: -0.01-0.35) |  |  |  | 35 | I | (?) |  |  |  |  |  |  |  |  |  |
| Strimpakos et al. (intra standing) | 35 | I | (-) (0.17-0.5) |  |  |  |  |  |  |  |  |  |  |  |  |  |  |  |
| Strimpakos et al. (inter) |  |  |  | 35 | I | (-) (ICC: -0.2-0.64) | 35 | I | (?) |  |  |  |  |  |  |  |  |  |
| Goncalves and Silva (intra within day HRNT) | 33 | D | (+) (ICC: 0.79-0.89) |  |  |  | 33 | D | (?) |  |  |  |  |  |  |  |  |  |
| Goncalves and Silva (intra within day TT) | 33 | D | (+) (ICC: 0.75-0.78) |  |  |  | 33 | D | (?) |  |  |  |  |  |  |  |  |  |
| Goncalves and Silva (intra within day F8T) | 33 | D | (+) (ICC: 0.83-0.93) |  |  |  | 33 | D | (?) |  |  |  |  |  |  |  |  |  |
| Goncalves and Silva (intra between day HRNT) | 33 | D | (+) (ICC: 0.75-0.85) |  |  |  | 33 | D | (?) |  |  |  |  |  |  |  |  |  |
| Goncalves and Silva (intra between day TT) | 33 | D | (-) (ICC: 0.57-0.59) |  |  |  | 33 | D | (?) |  |  |  |  |  |  |  |  |  |
| Goncalves and Silva (intra between day F8T) | 33 | D | (+) (ICC: 0.8-0.83) |  |  |  | 33 | D | (?) |  |  |  |  |  |  |  |  |  |
| Wibault et al. |  |  |  |  |  |  |  |  |  | 36 | D | (?) |  |  |  |  |  |  |
| Nikkhoo et al (within day intra US MOCAP) | 35 | D | (+) (ICC: 0.83-0.93) |  |  |  | 35 | I | (?) |  |  |  |  |  |  |  |  |  |
| Nikkhoo et al (between day intra US MOCAP) | 35 | D | (+) (ICC: 0.69-0.85) |  |  |  | 35 | I | (?) |  |  |  |  |  |  |  |  |  |
| Nikkhoo et al (within day intra IMU) | 35 | D | (+) (ICC: 0.66-0.91) |  |  |  | 35 | I | (?) |  |  |  |  |  |  |  |  |  |
| Nikkhoo et al (between day intra IMU) | 35 | D | (+) (ICC: 0.63-0.76) |  |  |  | 35 | I | (?) |  |  |  |  |  |  |  |  |  |
| Nikkhoo et al |  |  |  |  |  |  |  |  |  | 35 | A | (+) |  |  |  |  |  |  |
| Cid et al. | 28 | D | (-) (ICC: -0.16-0.5) |  |  |  |  |  |  |  |  |  |  |  |  |  |  |  |
| Kramer et al. (Intra) |  |  |  |  |  |  |  |  |  |  |  |  | 57 | D | (-) (ICC: 0.63) |  |  |  |
| Kramer et al. (inter) |  |  |  |  |  |  |  |  |  |  |  |  |  |  |  | 57 | D | (-) (ICC: 0.48) |
| Pooled or summary results | 537 | D | (+) (0.52-0.93) | 35 | I | (-) (ICC: -0.2-0.64) | 509 | I | (?) | 71 |  | (±) | 57 | D | (-) (ICC: 0.63) | 57 | D | (-) (ICC: 0.48) |
| GRADE | Very low (-2 risk of bias, -2 inconsistency) | | | Very low (-2 risk of bias, -1 imprecision) | | | Not graded | | | Not graded due to inconsistency | | | Very low (-3 risk of bias, -1 imprecision) | | | Very low (-3 risk of bias, -1 imprecision) | | |

**Healthy (AE) (THP) (Table 5 in manuscript)**

| Reference | Intrarater reliability | | | Measurement error | | |
| --- | --- | --- | --- | --- | --- | --- |
|  | n | Meth. Qual. | Rating | n | Meth. Qual. | Rating |
| Artz (intra within day sitting) | 21 | I | (-) (ICC: -0.81-0.66) | 21 | I | (?) |
| Artz (intra within day standing) | 21 | I | (-) (ICC: -0.11-0.68) | 21 | I | (?) |
| Artz (intra between day sitting) | 19 | I | (+) (ICC: -0.48-0.77) | 19 | I | (?) |
| Artz (intra between day standing) | 19 | I | (-) (ICC: 0.09-0.58)) | 19 | I | (?) |
| Kristjansson et al. 2001 (THP) | 19 | I | (+) (ICC: 0.69-0.74) | 19 | I | (?) |
| Goncalves and Silva (intra within day HR30T) | 33 | D | (+) (ICC: 0.78-0.83) | 33 | D | (?) |
| Goncalves and Silva (intra between day HR30T) | 33 | D | (+) (ICC: 0.55-0.76) | 33 | D | (?) |
| Pooled or summary results | 165 | I | (+) (-0.48-0.83) | 165 | I | (?) |
| GRADE | Very low (-2 risk of bias, -2 inconsistency) | | | Not graded | | |

**Healthy (CE) (NHP) (Table 6 in manuscript)**

| Reference | Intrarater reliability | | | Measurement error | | | Convergent validity | | | Criterion validity | | |
| --- | --- | --- | --- | --- | --- | --- | --- | --- | --- | --- | --- | --- |
|  | n | Meth. Qual. | Rating | n | Meth. Qual. | Rating | n | Meth. Qual. | Rating | n | Meth. Qual. | Rating |
| Lee et al. (NHP) | 20 | I | (+) (ICC: 0.38-0.84) | 20 | I | (?) |  |  |  |  |  |  |
| Dugailly et al. (90cm low speed) | 5 | I | (-) ICC: 0.22-0.47 | 5 | I | (?) |  |  |  | 17 | D | (?) |
| Dugailly et al. (90cm high speed) | 5 | I | (+) ICC: 0.58-0.79 | 5 | I | (?) |  |  |  |  |  |  |
| Dugailly et al. (180cm low speed) | 5 | I | (+) 0.52-0.75 | 5 | I | (?) |  |  |  |  |  |  |
| Dugailly et al. (180cm high speed) | 5 | I | (+) 0.8-0.86 | 5 | I | (?) |  |  |  |  |  |  |
| Dugailly (JPE vs disability) |  |  |  |  |  |  | 71 | A | (-) (r=0.32) |  |  |  |
| Dugailly (JPE vs pain intensity) |  |  |  |  |  |  | 71 | A | (-) (r=0.03) |  |  |  |
| Dugailly (JPE vs pain duration) |  |  |  |  |  |  | 71 | A | (-) (r=0.14) |  |  |  |
| Pooled or summary results | 40 | I | (+) ICC: 0.38-0.86 | 40 | I | (?) | 213 | A | (-) | 17 | D | (?) |
| GRADE (with reason) | Very low (-2 risk of bias, -2 imprecision, -2 inconsistency) | | | Not GRADED | | | Hight (multiple studies with adequate rating) | | | Very low (-3 risk of bias, -2 imprecision) | | |

**Healthy (CE) (THP) (Table 6 in manuscript)**

| Reference | Intrarater reliability | | | Measurement error | | |
| --- | --- | --- | --- | --- | --- | --- |
|  | n | Meth. Qual. | Rating | n | Meth. Qual. | Rating |
| Lee et al. (THP) | 20 | I | (+) (ICC: -0.47-0.83) | 20 | I | (?) |
| Pooled or summary results | 20 | I | (+) ICC: -0.47-0.83) | 20 | I | (?) |
| GRADE (with reason) | Very low (-3 risk of bias, -2 imprecision) | | | Not GRADED | | |
