# Supplementary figures and images for: Measurement properties of cervical joint position error in people with and without neck pain: a systematic review and narrative synthesis
Source: BMC Musculoskelet Disord. 2024 Jan 10;25:44. doi: 10.1186/s12891-023-07111-4 (PMC10777525; doi:10.1186/s12891-023-07111-4)

**Search example in Medline database**


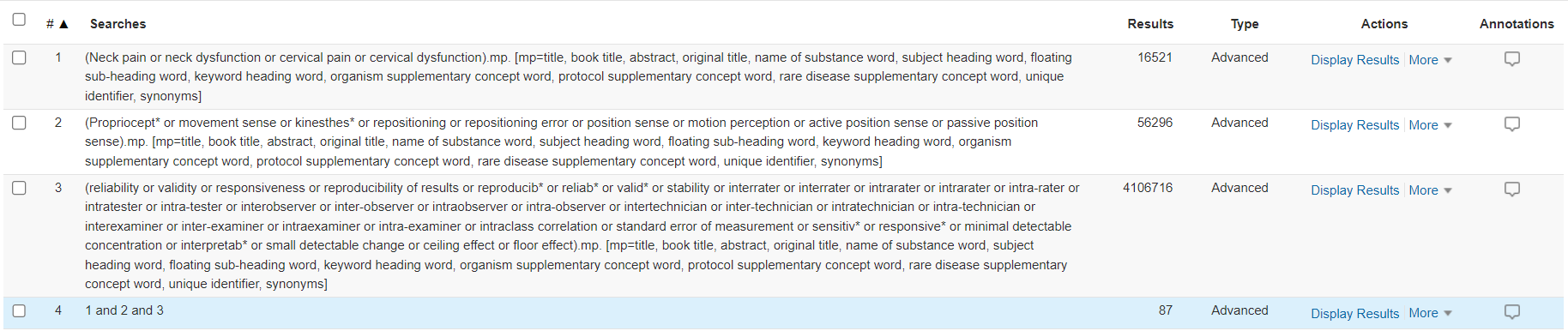

Supplement: Supplementary file 5 — Additional file 5. [file 12891_2023_7111_MOESM5_ESM.docx]
